# Large variability in the alkaloid content of *Corydalis yanhusuo* dietary supplements

**DOI:** 10.3389/fphar.2024.1518750

**Published:** 2025-01-15

**Authors:** Paula B. Luis, Claus Schneider

**Affiliations:** Department of Pharmacology and Vanderbilt Institute of Chemical Biology, Vanderbilt University School of Medicine, Nashville, TN, United States

**Keywords:** isoquinoline alkaloid, corydalis rhizome, tetrahydropalmatine, botanical extract, dietary supplement, consumer safety

## Abstract

**Introduction:**

Extracts from the rhizome of the traditional Chinese medicinal plant *Corydalis yanhusuo* (CY) mediate a number of biologic effects that are associated with its content of isoquinoline alkaloids. CY alkaloids have shown analgesic, cardioprotective, and anti-addictive effects in animal models of disease. Since CY alkaloids are available to consumers as dietary supplements we analyzed the content of alkaloids in 14 products including open powders, capsules, and liquid formulations, capturing a majority of the products available online in the US.

**Methods:**

Nineteen alkaloids were quantified using HPLC analyses with diode array detection after extraction using a weak cation exchange column.

**Results:**

Total alkaloid content was highly variable among the products, ranging from below quantifiable in some to ≈11 mg/g in others. Five of the products had comparable content of alkaloids (9.5 ± 1.6 mg/g), equaling about the amount of alkaloids of an extract prepared from CY rhizome (12.7 mg/g). The other samples had much lower content (1.8 ± 0.9 mg/g), or alkaloids were below quantifiable. One of the products was highly enriched in tetrahydropalmatine (≈5 mg/g), suggesting adulteration from the natural product, and raising concerns about possible toxicologic liability.

**Discussion:**

Considering alkaloid content as a key quality criterium for CY supplements, the large variability among the products seems unacceptable and makes it difficult for consumers to select products with an appropriate content of alkaloids.

## 1 Introduction

The plant *Corydalis yanhusuo* W.T. Wang (CY) is listed in the Chinese Pharmacopeia (Tian et al., 2020) and has been used in Traditional Chinese Medicine (TCM) to promote blood circulation, reinforce vital energy, and alleviate pain (Ma et al., 2008; Zhang et al., 2020). Biological effects of CY are mediated by its content of isoquinoline alkaloids of the protoberberine and tetrahydroprotoberberine structural classes. CY alkaloids were shown to have analgesic (Alhassen et al., 2021), cardioprotective and antiarrhythmic (Wu et al., 2007; Han et al., 2012; Li et al., 2022; Han et al., 2022), as well as anti-addictive effects (Chu et al., 2008; Wang and Mantsch, 2012; Nesbit and Phillips, 2020). For example, analgesic properties of CY have been associated with the alkaloids tetrahydropalmatine (Guo et al., 2014; Zhou et al., 2016; Kang et al., 2016; Liu et al., 2019) and dehydrocorybulbine (Zhang et al., 2014; Wang et al., 2016). A systematic analysis of a CY alkaloid extract showed effective attenuation of acute, inflammatory, and neuropathic pain in animal models without causing tolerance (Wang et al., 2016). Mechanistic studies showed that CY alkaloids were able to affect several pathways in the transmission of pain by targeting dopamine receptors (Wang et al., 2016), voltage gated sodium channels (Xu et al., 2021), NMDA and mGlu1/5 receptors (Dai et al., 2017), and the spinal sigma-1 receptor (Kang et al., 2016). The ability to modulate a number of different pain-related pathways in the absence of alkaloids with addiction potential makes CY an attractive candidate for a complementary-alternative approach towards pain management.

Toxicological and case studies indicate that CY alkaloids may not only mediate desirable effects but can also cause toxicity, mostly upon oral overdosing (Wu et al., 2021; Du et al., 2022). Overdosing with tetrahydropalmatine, one of the most abundant alkaloids of CY, in adult and pediatric populations resulted in depression of neurological, respiratory, and cardiac function as acute effects and chronic hepatitis after regular use (Lai and Chan, 1999). Case reports on overdosing, however, appeared related to consumption of products highly enriched in tetrahydropalmatine rather than using CY botanical extracts (Lai and Chan, 1999; Horowitz et al., 1996), indicating that toxicological consequences of consuming CY botanical extracts are unlikely yet not impossible.

Consumers might use CY dietary supplements as a source of bioactive alkaloids in order to address various ailments. The alkaloid content of CY dietary supplements is an important criterium for the quality of a product and its possible beneficial as well as toxicological effects. In order to understand what products are available to consumers and for researchers interested in using CY for animal or clinical research, we quantified alkaloids in CY dietary supplements, focusing on 19 abundant out of 84 reported alkaloids in CY (Feng et al., 2023).

## 2 Materials and methods

### 2.1 Materials

The following alkaloids were purchased from Cayman Chemical: scoulerine (item no. 35140; batch no. 0626850-3), isocorypalmine (item no. 29886; batch no. 0694012-1), glaucine (item no. 17338; batch no. 0481648-13), tetrahydropalmatine (item no. 20535; batch no. 0491304-7), tetrahydroberberine (item no. 33157; batch no. 0605003-10), corydaline (item no. 27654; batch no. 0570888-1), tetrahydrocoptisine (item no. 34603; batch no. 0620069-2), protopine (hydrochloride) (item no. 23366; batch no. 0514853-5), columbamine (item no. 35032; batch no. 0626484-3), coptisine (chloride) (item no. 28424; batch no. 0568598-14), palmatine (chloride) (item no. 30318; batch no. 0585590-3) and dehydrocorydaline (chloride) (item no. 30972; batch no. 0594357-5). CY dietary supplements and the CY rhizome sample (Plum Dragon Herbs) were ordered from online retail markets. Chemicals and HPLC solvents were from Sigma Aldrich or Fisher.

### 2.2 Large-scale alkaloid isolation and identification

CY dietary supplement sample 7 (2 g) was extracted with hot water (20 mL) by vortex mixing followed by centrifugation at 4,650 x g for 10 min. The supernatant was collected and the pellet was re-extracted with 10 mL of hot water. The supernatants were combined, centrifuged again (4,650 x g for 12 min) and loaded on a Waters Oasis WCX 6 cc cartridge (500 mg, 60 μm) that had been preconditioned with methanol and water. Two 2 g-aliquots of CY powder were extracted and processed in parallel. After loading the WCX cartridges were washed with 5% NH_4_OH (6 mL) and eluted with methanol (6 mL) followed by a solvent of acetonitrile/methanol/formic acid (80/20/2; by vol.; 6 mL) to give the WCX “methanol” and “formic acid” eluates, respectively. Preparation and elution of the WCX cartridge was performed according to the manufacturer’s instructions. The eluates were evaporated under a stream of N_2_ and dissolved in ca. 3 mL of methanol for the methanol eluate and ca. 150 μL of methanol for the formic acid eluate.

Semi-preparative RP-HPLC used an Agilent 1,200 Series HPLC system with a diode array detector and a Waters Symmetry C18 7 μm column (7.8 × 150 mm) eluted at 2 mL/min flow rate. For isolation of alkaloids of the methanol eluate the gradient used a solvent of water/acetonitrile/acetic acid 90/10/0.01 (by vol.) to 75/25/0.01 (by vol.) in 30 min, then changed to 20/80/0.01 (by vol.) in 2 min, hold 2 min, and return to starting solvent in 2 min. For the formic acid eluate the gradient used a solvent of 50 mM NH_4_OAc pH 8/acetonitrile 90/10 (by vol.) changed to 60/40 (by vol.) in 40 min, then changed to 30/70 (by vol.) in 2 min, hold 2 min, and return to starting solvent in 2 min. Use of a basic HPLC solvent for alkaloid purification was suggested by ref. (Zhang et al., 2009). Peaks from the formic acid eluate were collected in tubes that contained 50 μL of a mixture of water/acetic acid (4/1, by vol.) in order to prevent isolation artifacts occurring at basic pH (Marek et al., 2003).

The alkaloids collected from the methanol eluate were extracted from RP-HPLC solvent by evaporating acetonitrile under a stream of N_2_, adding water, and loading on a pre-conditioned Waters Oasis HLB 3 cc cartridge (60 mg). The cartridge was washed with water and alkaloids were eluted with methanol. Alkaloids from the formic acid eluate were extracted from RP-HPLC solvent by evaporation of acetonitrile and adding water and loading on a pre-conditioned Waters Oasis WCX 3 cc cartridge (60 mg). The cartridge was washed with water, and alkaloids were eluted with acetonitrile containing 1% acetic acid (750 μL) followed by acetonitrile (750 μL).

Collected alkaloids were checked for purity using a Waters Symmetry C18 5 μm column (4.6 × 250 mm) eluted at 1 mL/min flow rate with a gradient of water/acetonitrile/acetic acid 90/10/0.01 (by vol.) to 60/40/0.01 (by vol.) in 18 min. When isolated compounds had a purity (RP-HPLC UV 205 nm) less than 95% they were repurified. Re-purification was achieved using the same column and gradient as in the original purification or isocratic elution with a solvent of water/acetonitrile/acetic acid 85/15/0.01 (by vol.). Dehydrocorybulbine was re-purified using a gradient of 50 mM NH_4_OAc pH 8/acetonitrile 70/30 (by vol.) to 60/40 (by vol.) in 20 min; collected fractions were acidified using a mixture of water/acetic acid (4/1, by vol.) as described above.

### 2.3 Alkaloid extraction from dietary supplements and quantification

CY dietary supplements (25 mg) were weighed into a 2 mL Eppendorf tube. Hot water (ca 80°C; 1 mL) was added, spiked with internal standards (methyl nicotinate (MeN): 40 μg; nitidine chloride: 20 μg), and samples were vortex mixed for 1–2 min. After centrifugation (2 min at 17,000 x g) the supernatant was loaded on a pre-conditioned (3 mL of methanol followed by 3 mL of water) Waters Oasis WCX 3cc cartridge (60 mg, 30 μm). The cartridge was washed with 1 mL of 5% NH_4_OH and eluted with 1 mL of methanol followed by 1 mL of acetonitrile/methanol/formic acid (80/20/2, by vol.). Each supplement sample was extracted in two independent repeats. For the generation of calibration curves, different amounts of alkaloids were mixed with a fixed amount of standard (MeN or nitidine), and injected on RP-HPLC. Glaucine, tetrahydropalmatine, scoulerine, and corydaline standard curves were used to quantify alkaloids in the methanol eluate. Protopine, coptisine, and palmatine standard curves were used to quantify the alkaloids in the formic acid eluate.

For quantification an aliquot of 20 μL of each eluate (≈1 mL) was injected on RP-HPLC using an Agilent 1200 SL HPLC system equipped with a Water Symmetry Shield C18 5 μm column (4.6 × 250 mm) and a diode array detector with monitoring at 205, 220, 235, 270, 340, and 430 nm wavelength. The flow rate was 1 mL/min and compounds were eluted using a linear gradient of water/acetonitrile/acetic acid from 90/10/0.01 (by vol.) to 70/30/0.01 (by vol.) within 28 min followed by a wash step using water/acetonitrile/acetic acid 20/80/0.01 (by vol.). Alkaloids were quantified based on their peak area at 205, 220, or 340 nm.

### 2.4 CY rhizome extraction

CY rhizome was obtained in slices of 2–3 mm thickness and about 1 cm in diameter. Three slices were ground into a fine powder using a mortar and pestle. The powder (25 mg) was weighed into a 2 mL Eppendorf tube, spiked with internal standards, and alkaloids were extracted using 1 mL of hot water followed by WCX cartridge fractionation into methanol and formic acid eluates as described above.

### 2.5 HR-MS

High-resolution LC-MS of alkaloids was performed using a Q Exactive HF Hybrid Quadrupole-Orbitrap instrument (Thermo Fisher Scientific) operated in the electrospray ionization (ESI) positive mode. Prior to analysis, the instrument was calibrated with an ESI-positive ion calibration solution. A Waters Symmetry C18 column (2.1 × 50 mm, 1.8 µm) was eluted at room temperature with a gradient of acetonitrile in water/0.1% formic acid changed from 10% acetonitrile to 95% in 3 min at a flow rate of 0.4 mL/min.

### 2.6 NMR

Purified alkaloids were dissolved in deuterated solvents (CD_3_OD, CD_3_CN, CDCl_3_, or mixtures thereof) for NMR analysis. NMR spectra were recorded using a Bruker AV-II 600 MHz spectrometer equipped with a cryoprobe. Chemical shifts (δ value) are given relative to the residual non-deuterated solvent and are reported in parts per million (ppm). Coupling constants (*J*) are given in Hertz (Hz). Pulse frequencies were taken from the Bruker library.

## 3 Results

### 3.1 Selection of CY dietary supplements

A survey of three local health food and supplement stores in Nashville, TN conducted in March 2024 did not find any CY products available. Therefore, all CY dietary supplements were purchased online. Products containing CY in combination with other extracts or bioactive ingredients were excluded from the analysis. Fourteen different products were selected, capturing a majority of the products available. By comparison, the NIH/Office of Dietary Supplements Dietary Supplement Label Database (https://dsld.od.nih.gov/, accessed March 2024) listed about 13 different products on the market that contain CY as the only bioactive ingredient. Thus, the selected products provided a meaningful survey of CY dietary supplements available to consumers in the United States at the time.

The 14 selected products included open powders or granules (4), capsules (8), and two liquid formulations. Table 1 provides an overview of the product names, serving sizes, and supplement facts information retrieved from the labels. The “supplement facts” labels of samples 8, 10, and 12 identified the products as “Corydalis” yet not explicitly as “Corydalis yanhusuo”. Sample 8 was referred to as “Yan Hu Suo” elsewhere on the label, implying that the product contained an extract of CY. The two other samples (10 and 12) were assumed to contain CY, or might be mistaken by consumers to do so, and thus were included in the analysis.

**TABLE 1:** CY dietary supplements included in the analysis.

Sample no.	Serving size	Form	“Supplement facts” label
1	1,000 mg	capsule	Corydalis root P.E. 10:1 *(Corydalis Yanhusuo)*
2	1,000 mg	powder	Corydalis Extract *(Corydalis Yanhusuo)* (Root)
3	500 mg	capsule	Corydalis Extract (Corydalis yanhusuo) (Root) (A 20:1 extract equivalent to 10,000 mg of Corydalis root powder)
4	1,000 mg	capsule	Corydalis Root P.E. 10:1 (Corydalis Yanhusuo)
5	400 mg	capsule	*Corydalis yanhusuo* (rhizome)
6	500 mg	capsule	Corydalis (15:1 Extract) (Corydalis Yanhusuo) (Root)
7	1,000 mg	powder/granules	Corydalis yanhusuo (tuber) (100 g of the concentrated granules extracted from 500 g of the raw herbs)
8	1,000 mg	capsule	Corydalis Extract (4:1 Hot Water Extract)^a^
9	1,000 mg	capsule	Corydalis Extract *(Corydalis yanhusuo)* (rhizome) (DHCB dehydrocorybulbine)
10	1,000 mg	capsule	Corydalis Root 10:1 (Root) Extract (equivalent to 10,000 mg)
11	1,000 mg	powder	Corydalis root (yan hu suo)
12	not given	powder	Corydalis poppy powder^b^
13	0.7 mL	liquid	Corydalis tuber (*Corydalis yanhusuo*) (purity verified) (extract 665 mg) Extraction rate 233 mg herb per 0.7 mL. Dry herb:menstruum ratio 1:3
14	5 drops	liquid	Corydalis Root (Corydalis yanhusuo)^c^

^a^ Elsewhere on the label there is a reference to “Yan Hu Suo”.

^b^ Package does not have a “Supplement Facts” label.

^c^ Labeled as “Product Information” instead of “Supplement Facts”.

### 3.2 Alkaloid identification

**FIGURE 1:**
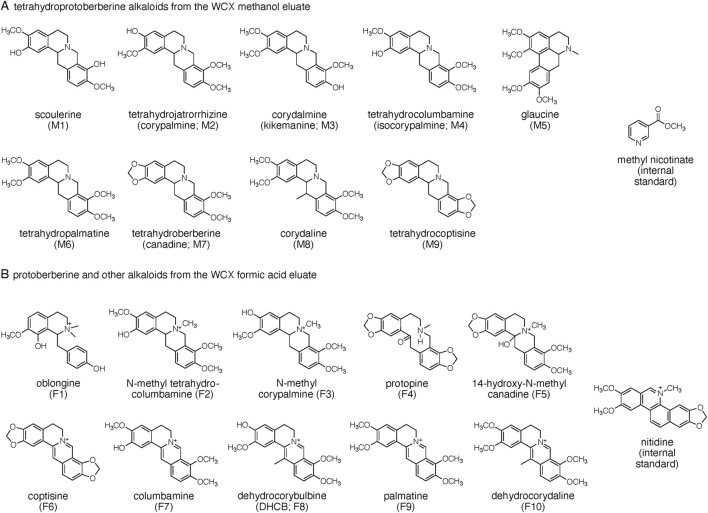
Structures of alkaloids quantified in the CY dietary supplements. Alkaloids are grouped into **(A)** tetrahydroprotoberberines and **(B)** protoberberines and others as obtained by differential elution from the weak cation exchange (WCX) cartridge. Methyl nicotinate and nitidine were used as internal standards for quantification of the respective alkaloid groups.

We isolated and identified 19 alkaloids from one of the CY dietary supplements (sample 7 in Table 1) that had shown high alkaloid content in a preliminary analysis. Alkaloids were solubilized using hot water and separated from other material by binding to a weak cation exchange (WCX) cartridge. Alkaloids were eluted from the WCX cartridge using methanol to obtain tertiary amine alkaloids (i.e., tetrahydroprotoberberines; “methanol eluate”) followed by elution with a solvent of acetonitrile/methanol/formic acid (80/20/2, by vol.) to provide mostly quaternary amine alkaloids (i.e., protoberberines and others; “formic acid eluate”). Alkaloids were purified using semi-preparative RP-HPLC; structural identification of isolated alkaloids was achieved using NMR and HR-MS analyses and/or comparison to authentic standards when available (see Supplementary Material). The structures of nine alkaloids (M1-M9) isolated from the methanol and 10 alkaloids (F1-F10) from the formic acid WCX eluates, respectively, are shown in Figure 1.

### 3.3 Analytical approach for alkaloid quantification

**FIGURE 2:**
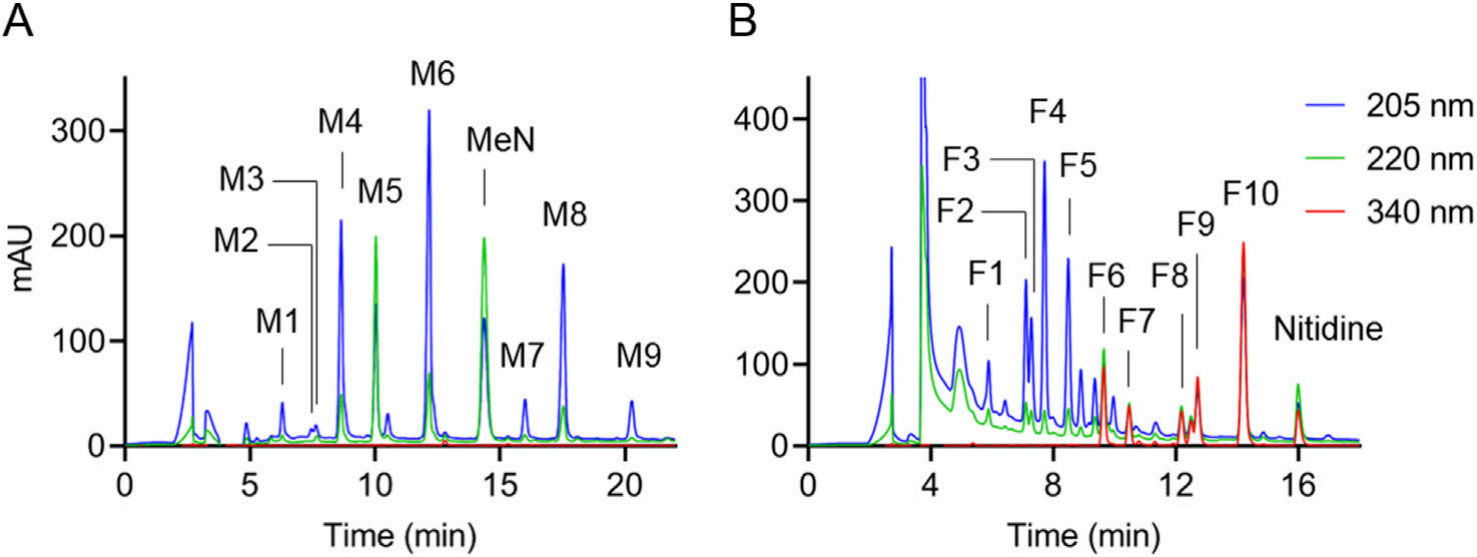
Analysis of alkaloids in CY dietary supplement 7, representative of a CY dietary supplement with high content of alkaloids. RP-HPLC analysis with UV/Vis diode array detection of the **(A)** methanol and **(B)** formic acid eluates from the WCX cartridge used to enrich alkaloids after hot water extraction of the sample. The sample was spiked with internal standards methyl nicotinate (MeN) and nitidine prior to extraction. Peak labels refer to the numbering of alkaloids in Figure 1. The chromatograms were recorded at 205 nm (blue), 220 nm (green), and 340 nm (red).

Alkaloids were solubilized from the dietary supplements using hot water and applied to a WCX column to provide a “methanol” and “formic acid” eluate for separate RP-HPLC analysis. The method used for quantification of alkaloids is illustrated in Figure 2, showing RP-HPLC analysis with UV/Vis diode array detection of sample 7 as example. Both the tertiary (methanol eluate; Figure 2A) and quaternary (formic acid eluate; Figure 2B) amine alkaloids obtained from the WCX cartridge were well resolved. Resolution and peak shape of alkaloids was enhanced by using a Symmetry Shield C18 column (Waters) that provides embedded polar carbamate groups at the base of each ligand. The embedded polar groups bind water molecules that “shield” charged silanol groups and therefore decrease the amount of tailing with charged basic compounds. Regardless of what HPLC column was used we noticed that alkaloids showed a tendency to switch elution order and change retention times beyond what was expected upon variations in chromatographic conditions, even when variations were minor. Similar effects were observed when different amounts of alkaloids were injected. Thus, caution should be used in peak identification especially since the UV spectra of many of the alkaloids are similar. Two internal standards, methyl nicotinate and nitidine, were added to the supplements for quantification of alkaloids in the WCX methanol and formic acid eluates, respectively. The standards were present only in the intended WCX eluates, were well resolved from the alkaloids of interest, had suitable UV/Vis spectra, and were absent from a list of known alkaloids of CY (Feng et al., 2023).

Alkaloids were grouped by their UV/Vis spectra for quantification. Alkaloids had one of two general types of UV/Vis spectra, based on the degree of saturation in the third ring. Protoberberines are yellow in color with several maxima around 230 nm, 270 nm, 340 nm, and 430 nm whereas the saturated tetrahydroprotoberberines have mostly end absorbance in the UV and are lacking color (Supplementary Figures 1, 2). Accordingly, alkaloids were quantified using the absorbance of the chromatographic peaks at 205 nm, 220 nm, or 340 nm as appropriate. For the methanol eluate, a calibration curve for scoulerine (M1) was used to quantify M1, M2, M3, and M4 at 205 nm. Glaucine (M5) and tetrahydropalmatine (M6) calibration curves were used to quantify M5 and M6, respectively, at 220 nm. A calibration curve for corydaline (M8) was used to quantify M7, M8, and M9 at 205 nm. For alkaloids in the formic acid eluate, a calibration curve for protopine (F4) was used to quantify F1, F2, F3, F4, and F5 at 205 nm. A calibration curve for coptisine (F6) was used to quantify F6, F7, and F8 at 340 nm. A calibration curve for palmatine (F9) was used to quantify F9 and F10 at 340 nm (see Supplementary Material). The LOQ was between 5 and 6 ng per injection (20 μL) on HPLC.

### 3.4 Content of alkaloids in CY dietary supplements

**FIGURE 3:**
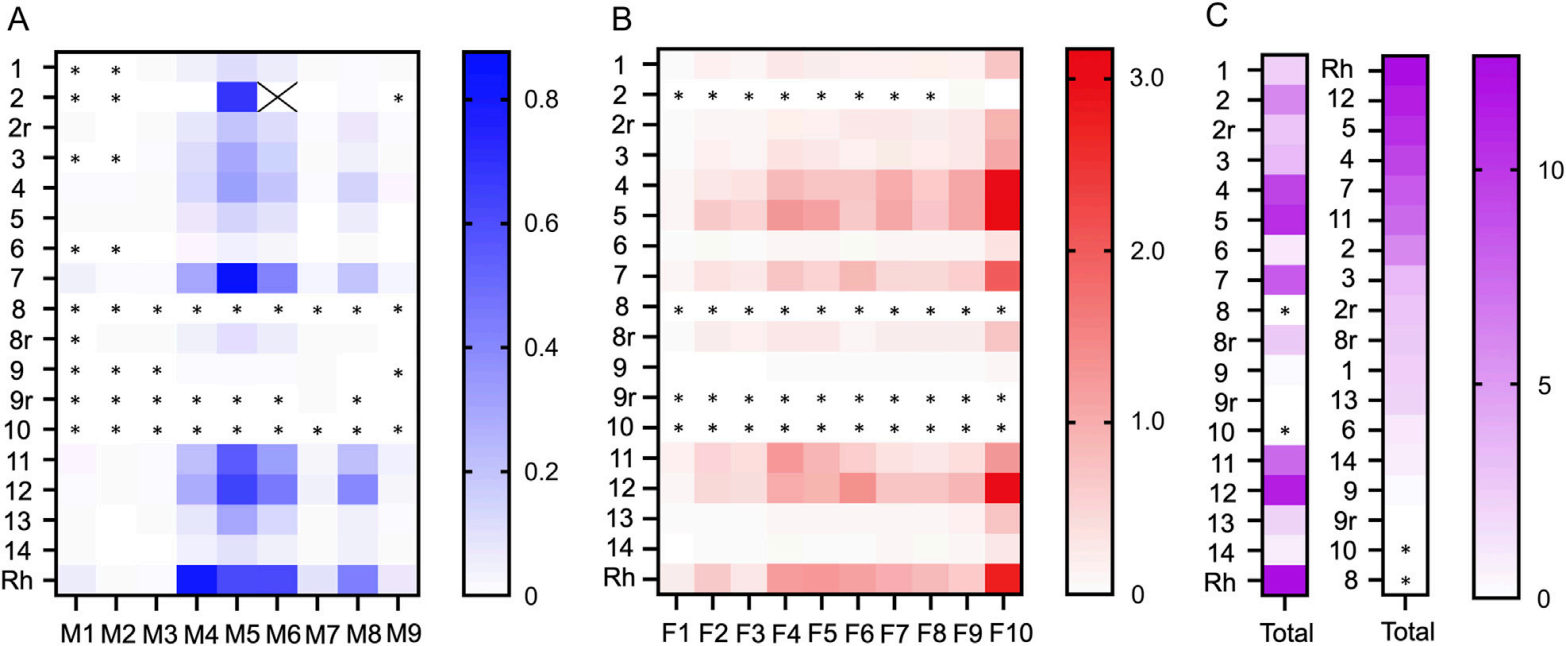
Graphic representation of the alkaloid content in the CY dietary supplements. The colored matrices show the content of alkaloids (mg/g) of the **(A)** methanol eluate (M1-M9) and **(B)** formic acid eluate (F1-F10) for dietary supplement samples 1-14 and *Corydalis yanhusuo* rhizome (Rh). **(C)** The total sum of alkaloids (mg/g) for each supplement is shown, sorted by sample number in the left column and by amount in the middle column. Samples marked with a lowercase “r” were repurchased. Alkaloid content in the supplements is coded by color intensity. The “X” in panel A denotes tetrahydropalmatine in sample 2 that was omitted from the graph because the concentration was 6-fold higher than the next most abundant alkaloid. Alkaloids that were below LOQ are marked with an asterisk (*).

A graphical summary of the quantification results is shown in Figure 3. The content of alkaloids in the methanol and formic acid eluates of the CY dietary supplements is listed in Tables 2, 3, respectively. The most abundant alkaloids in the CY dietary supplements were tetrahydrocolumbamine (M4), glaucine (M5), tetrahydropalmatine (M6), corydaline (M8), protopine (F4), 14-hydroxy-N-methylcanadine (F5), coptisine (F6), and dehydrocorydaline (F10) (Figures 3A, B). Based on their content of alkaloids the CY dietary supplements roughly fell into three groups (Figure 3C). A group of five supplements (samples 4, 5, 7, 11, 12) had an overall high and similar content of alkaloids (9.5 ± 1.6 mg/g). The remaining samples either had a much lower content of alkaloids (samples 1, 3, 6, 13, 14; 1.8 ± 0.9 mg/g), or the alkaloids were near or below the lower limit of quantification (samples 8, 9, 10). When a 4-fold higher amount of the latter three samples was extracted the results were unchanged.

**TABLE 2:** Alkaloid content (mean ± SD in ng/mg) in the WCX methanol eluate of the CY dietary supplements. Alkaloids are identified by name and numbering used in Figure 1 as well as the wavelength used for quantification. Missing entries indicate values were below the LOQ. Samples marked with a lowercase “r” were repurchased. Rh, *Corydalis yanhusuo* rhizome.

Sample ID	Scoulerine (M1; 205 nm)	Tetrahydro-jatrorrhizine (M2; 205 nm)	Corydalmine (M3; 205 nm)	Tetrahydro-columbamine (M4; 205 nm)	Glaucine (M5; 220 nm)	Tetrahydro-palmatine (M6; 220 nm)	Tetrahydro-berberine (M7; 205 nm)	Corydaline (M8; 205 nm)	Tetrahydro-coptisine (M9; 205 nm)	Total
1			4.3 ± 0.12	46.4 ± 2.49	103.2 ± 4.66	56.8 ± 2.01	4.3 ± 0.02	16.3 ± 1.41	4.3 ± 0.00	236 ± 10.73
2			2.5 ± 0.27	0.9 ± 1.26	686.3 ± 27.70	5,217.1 ± 247.86	2.1 ± 0.31	6.9 ± 0.63		5,916 ± 275.51
2 r	6.2 ± 0	3.2 ± 0.12	3.8 ± 0.10	77.8 ± 0.60	196.1 ± 1.03	110.0 ± 1.23	8.2 ± 0.87	68.6 ± 0.73	12.0 ± 0.80	486 ± 4.04
3			7.3 ± 0.05	118.5 ± 6.80	293.4 ± 12.42	149.3 ± 5.77	6.1 ± 0.99	51.6 ± 2.98	4.7 ± 1.26	631 ± 30.17
4	9.3 ± 0.09	9.3 ± 2.95	5.6 ± 0.22	128.6 ± 1.14	325.6 ± 10.86	188.1 ± 6.04	16.1 ± 1.08	142.9 ± 3.87	18.6 ± 1.24	844 ± 21.15
5	5.7 ± 0.07	4.2 ± 0.12	3.5 ± 0.99	69.4 ± 2.23	142.1 ± 6.55	86.0 ± 4.81	2.7 ± 0.17	54.6 ± 3.06	1.2 ± 1.05	369 ± 16.59
6			1.9 ± 0.12	17.8 ± 0.82	39.1 ± 1.26	23.7 ± 0.83	1.3 ± 0.07	5.7 ± 0.08	1.4 ± 0.11	91 ± 2.53
7	36.1 ± 1.00	9.3 ± 1.04	16.4 ± 0.00	299.5 ± 1.40	876.9 ± 11.71	421.0 ± 13.85	31.3 ± 0.22	201.3 ± 1.26	34.2 ± 0.30	1926 ± 28.35
8										
8 r		4.3 ± 6.10	3.7 ± 0.10	37.0 ± 1.19	102.2 ± 1.84	51.8 ± 1.64	3.5 ± 0.19	4.3 ± 0.58	1.6 ± 0.02	208 ± 0.79
9				8.6 ± 0.19	16.3 ± 0.23	7.3 ± 0.23	3.6 ± 0.19	3.4 ± 0.10		39 ± 0.56
9 r							3.7 ± 0.02		2.0 ± 0.28	6 ± 0.30
10										
11	18.2 ± 0.44	6.6 ± 1.74	9.7 ± 0.90	219.5 ± 5.56	558.9 ± 11.11	323.3 ± 13.58	29.3 ± 0.29	219.9 ± 4.16	37.9 ± 0.76	1,423 ± 34.17
12	14.6 ± 0.00	5.6 ± 0.90	9.3 ± 0.58	279.9 ± 3.41	646.4 ± 0.53	451.7 ± 21.26	34.7 ± 0.34	404.0 ± 5.42	24.0 ± 0.71	1870 ± 28.09
13	4.9 ± 0.19	2.4 ± 0.19	3.6 ± 0.41	81.1 ± 3.07	300.5 ± 1.28	127.4 ± 1.46	6.0 ± 0.17	50.8 ± 0.21	8.4 ± 0.16	585 ± 5.67
14	4.0 ± 0.23	1.0 ± 1.40	2.8 ± 0.05	40.3 ± 0.99	97.1 ± 4.94	47.7 ± 4.16	4.3 ± 0.58	42.5 ± 1.88	4.0 ± 0.09	244 ± 13.86
Rh	54.1 ± 2.11	6.6 ± 2.40	12.2 ± 0.53	820.1 ± 35.78	616.4 ± 24.41	621.3 ± 31.76	85.6 ± 3.87	432.6 ± 20.21	70.9 ± 2.22	2,720 ± 123.29

**TABLE 3:** Alkaloid content (mean ± SD in ng/mg) in the WCX formic acid eluate of the CY dietary supplements. Alkaloids are identified by name and numbering used in Figure 1 as well as the wavelength used for quantification. Missing entries indicate values were below the LOQ. Samples marked with a lowercase “r” were repurchased. Rh, *Corydalis yanhusuo* rhizome.

Sample ID	Oblongine (F1; 205 nm)	N-Methyltetrahy-drocolumbamine (F2; 205 nm)	N-Methyl-corypalmine (F3; 205 nm)	Protopine (F4;205 nm)	14-Hydroxy-N-methylcanadine (F5; 205 nm)	Coptisine (F6; 340 nm)	Columbamine (F7; 340 nm)	Dehydro-corybulbine (F8; 340 nm)	Palmatine (F9; 340 nm)	Dehydro-corydaline (F10; 340 nm)	Total
1	48 ± 0.9	142 ± 4.9	112 ± 2.2	272 ± 12.8	192 ± 6.6	165 ± 4.6	147 ± 4.0	175 ± 3.9	158 ± 4.3	677 ± 15.8	2088 ± 55.5
2									61 ± 1.4	10 ± 0.1	71 ± 1.5
2 r	19 ± 0.3	93 ± 3.4	73 ± 2.9	179 ± 0.5	132 ± 1.6	249 ± 1.9	269 ± 2.2	209 ± 1.3	296 ± 1.8	922 ± 4.2	2,440 ± 13.3
3	47 ± 5.2	149 ± 15.8	120 ± 11.6	318 ± 20.2	249 ± 30.1	171 ± 11.4	237 ± 22.4	236 ± 23.1	260 ± 27.7	1,049 ± 108.3	2,835 ± 275.8
4	83 ± 23.6	287 ± 64.1	317 ± 89.6	842 ± 224.4	715 ± 184.1	685 ± 102.7	1,020 ± 155.1	618 ± 97.5	1,058 ± 162.2	3,079 ± 477.4	8,703 ± 1,452.7
5	124 ± 12.1	639 ± 76.8	505 ± 78.3	1,272 ± 150.8	1,102 ± 131.7	649 ± 93.5	1,076 ± 144.5	665 ± 88.5	1,094 ± 146.3	3,173 ± 416.0	10,301 ± 1,338.5
6	16 ± 1.2	58 ± 1.7	47 ± 1.6	108 ± 3.3	94 ± 1.1	53 ± 0.7	84 ± 1.9	74 ± 0.3	92 ± 0.5	321 ± 2.8	948 ± 8.2
7	108 ± 9.7	341 ± 10.7	261 ± 7.5	693 ± 16.0	523 ± 17.4	867 ± 32.1	482 ± 10.3	463 ± 4.7	571 ± 7.2	1994 ± 28.8	6,303 ± 144.4
8											
8 r	21 ± 7.8	196 ± 49.2	154 ± 40.7	254 ± 57.8	262 ± 61.4	124 ± 14.0	216 ± 24.8	193 ± 22.2	218 ± 24.9	695 ± 81.3	2,334 ± 368.4
9	4 ± 0.1	12 ± 0.3	10 ± 0.3	20 ± 0.1	17 ± 0.2	16 ± 0.2	18 ± 0.5	13 ± 0.3	20 ± 0.3	64 ± 0.9	194 ± 2.9
9 r											
10											
11	159 ± 41.8	491 ± 119.7	374 ± 98.6	1,252 ± 268.4	919 ± 200.2	600 ± 6.0	340 ± 0.2	287 ± 0.2	377 ± 0.2	1,265 ± 3.3	6,067 ± 718.8
12	82 ± 10.3	461 ± 78.4	405 ± 72.3	1,021 ± 159.7	932 ± 151.5	1,356 ± 216.0	690 ± 124.7	697 ± 131.3	883 ± 167.8	3,032 ± 569.2	9,558 ± 1,681.2
13	28 ± 0.4	45 ± 0.0	36 ± 0.4	106 ± 1.2	87 ± 1.1	68 ± 1.0	124 ± 1.0	103 ± 0.6	140 ± 1.4	715 ± 6.1	1,451 ± 12.4
14	9 ± 0.1	26 ± 0.2	20 ± 0.3	53 ± 0.9	43 ± 0.5	44 ± 0.8	75 ± 2.2	52 ± 1.9	67 ± 2.5	279 ± 9.7	668 ± 18.5
Rh	188 ± 1.2	657 ± 28.3	286 ± 14.7	1,228 ± 36.1	1,249 ± 58.2	1,127 ± 69.8	982 ± 63.9	854 ± 69.7	611 ± 46.8	2,758 ± 200.8	9,939 ± 587.1

**FIGURE 4:**
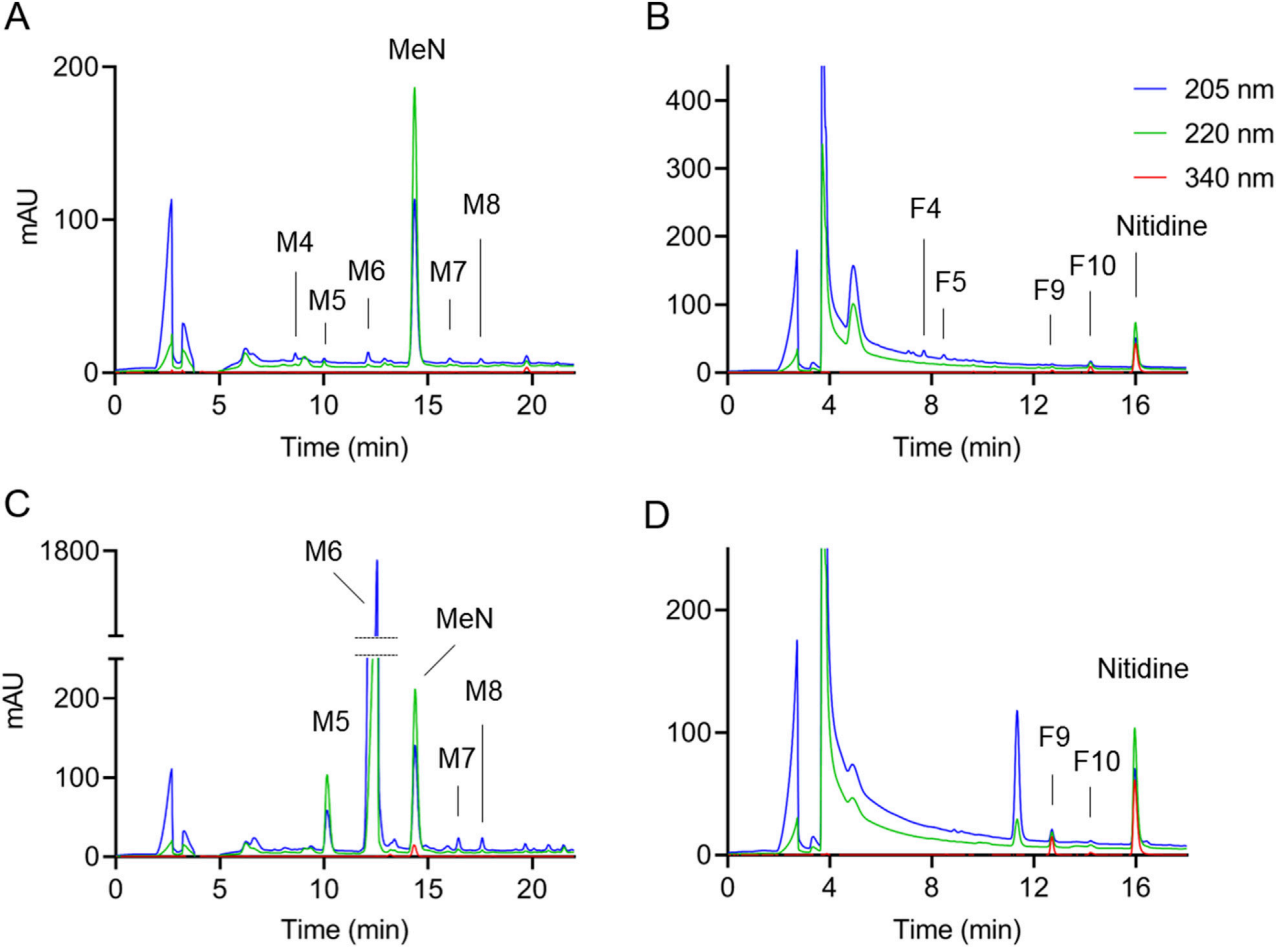
Analysis of CY dietary supplements with low or unusual content of alkaloids. RP-HPLC analysis of samples 9 (top) and 2 (bottom) of the **(A, C)** methanol and **(B, D)** formic acid WCX eluates, respectively. Peak labels refer to the numbering of alkaloids in Figure 1. The chromatograms were recorded at 205 nm (blue), 220 nm (green), and 340 nm (red). MeN, methyl nicotinate.

The near absence of the alkaloids of interest is illustrated for sample 9 (Figures 4A, B), showing RP-HPLC chromatograms of the methanol and formic acid eluates of the WCX column. No other peaks with UV/Vis spectra characteristic of isoquinoline alkaloids were detected, indicating that the sample was largely devoid of this type of bioactive alkaloids. For sample 2 the unusually high content of tetrahydropalmatine together with an overall low to absent content of other alkaloids is shown in Figures 4C, D. Tetrahydropalmatine was present in sample 2 at about 5 mg/g (i.e., per suggested serving size), which was >10-times the amount of tetrahydropalmatine in the next highest sample. The fact that only one alkaloid was of high abundance in the sample suggested it was added as a pure compound (of synthetic or natural origin) and was not included as an extract from another botanical which would likely have provided additional alkaloids.

Three of the four samples with unusual or low to absent alkaloid content (samples 2, 8, and 9; sample 10 was no longer available) were repurchased in order to test a different batch for consistency of the findings. In repurchased sample 2 the excessive content of tetrahydropalmatine was no longer present while other alkaloids were increased. Sample 8, which did not contain any alkaloids in the original sample, showed an increased though low amount in the repurchased sample. For sample 9, which had very low content of alkaloids, the amount was even further decreased, and the repurchased sample contained only three alkaloids that were barely above the LOQ. Even though the product label explicitly mentioned “DHCB” (i.e., dehydrocorybulbine) sample 9 had the lowest content of dehydrocorybulbine of all samples, and the amount was decreased to below LOQ in the repurchased sample.

### 3.5 Comparison to CY rhizome

The alkaloids of the CY dietary supplements were compared to an alkaloid extract prepared from the rhizome of CY. The alkaloids in the supplements with high alkaloid content and in the CY rhizome were similar regarding type and their relative abundance (Figure 3). Quantitative analysis showed that the absolute amount of alkaloids in the supplements with high content of alkaloids (9.5 ± 1.6 mg/g) was similar to the rhizome (12.7 mg/g) (Tables 2, 3).

## 4 Discussion

*In vitro* and animal studies testing the biologic effects of CY alkaloids most often use a lab-prepared extract from the rhizome or chemically synthesized or isolated compounds. For consumers interested in exploring the potential health benefits of CY these sources are not readily accessible and they are instead likely to rely on CY dietary supplements as a source of the respective alkaloids. CY dietary supplements are labeled as containing extracts from CY rhizome, but which alkaloid and how much of each is present in a given CY dietary supplement is not listed on the product label (cf. Table 1). To our knowledge, the alkaloid content of CY dietary supplements has not been analyzed and made publicly available, and it is not known whether CY dietary supplements provide an adequate source of bioactive alkaloids.

We have developed a targeted analytical method to quantify CY alkaloids based on their UV/Vis absorbance upon resolution of the compounds by RP-HPLC following extraction on a WCX column. The HPLC-UV/Vis analysis (cf. Figures 2, 4) also provides a visual impression of the relative abundance of alkaloids, normalized by the internal standards, that is representative of the actual content since the molar absorption coefficients are similar within the groups of saturated and unsaturated alkaloids, respectively, (Shammna et al., 1969). The approach was suitable to clearly identify and quantify 19 of the most abundant alkaloids in the CY supplements, and to document their (near) absence in others. While it is possible that the CY supplements contained additional alkaloids not assessed via this approach, these were low in abundance and thus less likely to be biologically relevant. Our quantification method using WCX extraction combined with HPLC and diode array detection provides a simple, sensitive, reliable, and affordable approach for the analysis of alkaloids in CY dietary supplements that can be readily implemented for product control.

A limitation to our approach is that the developed analytical method was not validated. While we are convinced that the lack of validation does not compromise the overall findings on the alkaloid content in the dietary supplements, we want to emphasize a key aspect for the method to be reproduced by other laboratories. We found that especial attention needs to be placed on the correct identification of alkaloid peaks when even seemingly minor modifications are made to chromatographic parameters (solvent composition, gradient), or when switching between HPLC columns of the identical type. For the alkaloids with closely related UV/Vis spectra changes in retention times or elution order may be difficult to spot, and ideally should be confirmed by comparison to authentic standards.

We found an unexpectedly large range in the content of alkaloids of different CY dietary supplements. Only about one-third of the CY dietary supplements (5 out of 14; samples 4, 5, 7, 11, 12) provided what can be considered an acceptable amount of alkaloids when compared to the type and amount of alkaloids obtained from a CY rhizome sample. A second group of supplements (samples 1, 3, 6, 13, 14) had a considerably lower content of alkaloids, and in the remaining four samples (2, 8, 9, 10) alkaloids were either below quantifiable, low, or inconsistently present when a different batch was analyzed. There was no correlation between the form of the supplement (powder, capsule, liquid) and the content of alkaloids.

Type and relative amount of alkaloids in the supplements with high alkaloid content were similar to what was present in a CY rhizome sample analyzed here and as described in refs. (Ma et al., 2008; Ding et al., 2007). Variability of alkaloid content in response to differences in growing conditions appears to be small relative to the variability in the supplement products observed here (Lu et al., 2020; Chen et al., 2020). It does not seem technically justified then that so many of the supplements had a very low alkaloid content, considering that the process for making CY dietary supplements most likely involves grinding of the rhizome into a powder followed by a simple extraction of the ground rhizome with hot water.

One of the products with an overall low amount of alkaloids (sample 2) stood out due to its high content of tetrahydropalmatine, a cause for concern regarding adulteration as well as potential toxicologic liability (Du et al., 2022; Lai and Chan, 1999). This sample provided about 5 mg tetrahydropalmatine per serving which is about 5-fold below the content of tablets that were causing acute poisoning as a result of unregulated use of biopharmaceuticals containing purified tetrahydropalmatine (Lai and Chan, 1999). In these cases of overdosing, two of the patients were assumed to have consumed up to 1,500 mg and 2000 mg of tetrahydropalmatine, respectively, while the recommended safe daily oral doses were quoted as 60–180 mg (Wang and Mantsch, 2012; Lai and Chan, 1999). While it appears unlikely that consumers would ingest an amount of sample two that might result in toxic exposure to tetrahydropalmatine, it cannot be safely excluded. The high content of tetrahydropalmatine in sample two was most likely due to adulteration whereas the content in the repurchased sample was within the range of other samples. It should be noted that due to safety concerns, certain preparations containing tetrahydropalmatine are banned by the US FDA for human consumption in the US (Horowitz et al., 1996; Woolf et al., 1994).

Two of the three samples that were essentially devoid of alkaloids (samples 8 and 10) were labeled as “Corydalis” – i.e., not as “Corydalis yanhusuo”. This could indicate the particular botanical present in the supplement was not CY but possibly another *Corydalis* species, e.g., *Corydalis ambigua*. While this might explain the (near) absence of typical CY alkaloids, there was no indication from our analyses that the samples contained other alkaloids nor did the label suggest that another *Corydalis* species was present. It seems unlikely that consumers would pick up on the difference in the name and change expectations accordingly. Most likely, consumers expect supplements labeled as “Corydalis” to contain a CY extract, similar to us when we selected the products for analysis. This rationale is supported by the fact that sample 12, labeled as “Corydalis poppy powder”, had among the highest content of typical CY alkaloids even though the label did not include the term “yanhusuo”. Thus, the supplements containing a nearly undetectable or inconsistent amount of alkaloids appear fraudulent even though some of them may not be in strictly legal terms due to the incomplete naming.

The content of alkaloids in CY dietary supplements is a key measure for quality of the products besides any markers of safety–that were not assessed in the current study. The large range in alkaloid content between the products makes it difficult for consumers to find one that meets their expectations regarding product quality, i.e., alkaloid content. A large fraction of the available products may not meet consumer expectations, and when a product does not deliver an expected biologic effect it might be due to the lower than expected content of bioactive alkaloids. Consumers unsure about which CY dietary supplement to select may want to try different products or might consider using the rhizome since our analyses suggested that the content of alkaloids in the ground CY rhizome exceeds that of any supplement, and in addition there is less opportunity for adulteration. For researchers interested in testing CY in animal or human studies it is advisable to conduct a thorough chemical analysis of the product intended for use, a requirement that extends to all clinical and non-clinical studies with botanical products (Hosbas Coskun et al., 2021; Funk and Schneider, 2021; Sorkin et al., 2022; Heinrich et al., 2022).

We have previously participated in a study aimed at analyzing turmeric dietary supplements with regard to curcuminoid content and safety parameters (Skiba et al., 2018). The study found that turmeric dietary supplements in the US marketplace largely contained the declared amount of curcuminoids and were safe with respect to residual solvents from the extraction process and free from lead contamination. Turmeric/curcumin is a top selling dietary supplement in the US and around the world (Panknin et al., 2023; Skiba et al., 2020), and the size of the market may contribute to an overall consistent quality as well as efficient monitoring. CY dietary supplements, in contrast, are more of a niche product, and this may underlie current deficiencies in content and control of available products. An overview on the current status of adulteration of botanical products focusing on five major ingredients including turmeric can be found in ref. (Orhan et al., 2024).

Our analysis of alkaloid content in CY dietary supplements has established a baseline profile and amount of alkaloids equivalent to the natural product that was present in about one-third of the products. We have uncovered substantial deficiencies in this product segment available to US customers online. The large variability in alkaloid content between and within different products, with some appearing fraudulent or substantially adulterated from the natural product, seems unacceptable. The excessive amount of tetrahydropalmatine in one of the products was alarming and possibly a health concern. The shortcomings are unlikely due to variability in the natural product used as source. In the current marketplace consumers and researchers alike should be aware that not all CY dietary supplements may meet expectations regarding key quality criteria.

## Data Availability

The original contributions presented in the study are publicly available. This data can be found here: https://osf.io/b68ps/.

## Abbreviations

CY, Corydalis yanhusuo; DHCB, dehydrocorybulbine; HR-MS, high resolution-mass spectrometry; LOQ, limit of quantification, MeN, methyl nicotinate, ODS, Office of Dietary Supplements; WCX, weak cation exchange.
