# Supplementary material for: Large variability in the alkaloid content of Corydalis yanhusuo dietary supplements
Source: Front Pharmacol. 2025 Jan 15;15:1518750. doi: 10.3389/fphar.2024.1518750 (PMC11774941; doi:10.3389/fphar.2024.1518750)
Supplement: Supplementary file 1 [file DataSheet1.docx]

Supplementary Material

# Supplementary Figures and Tables

## Supplementary Figures


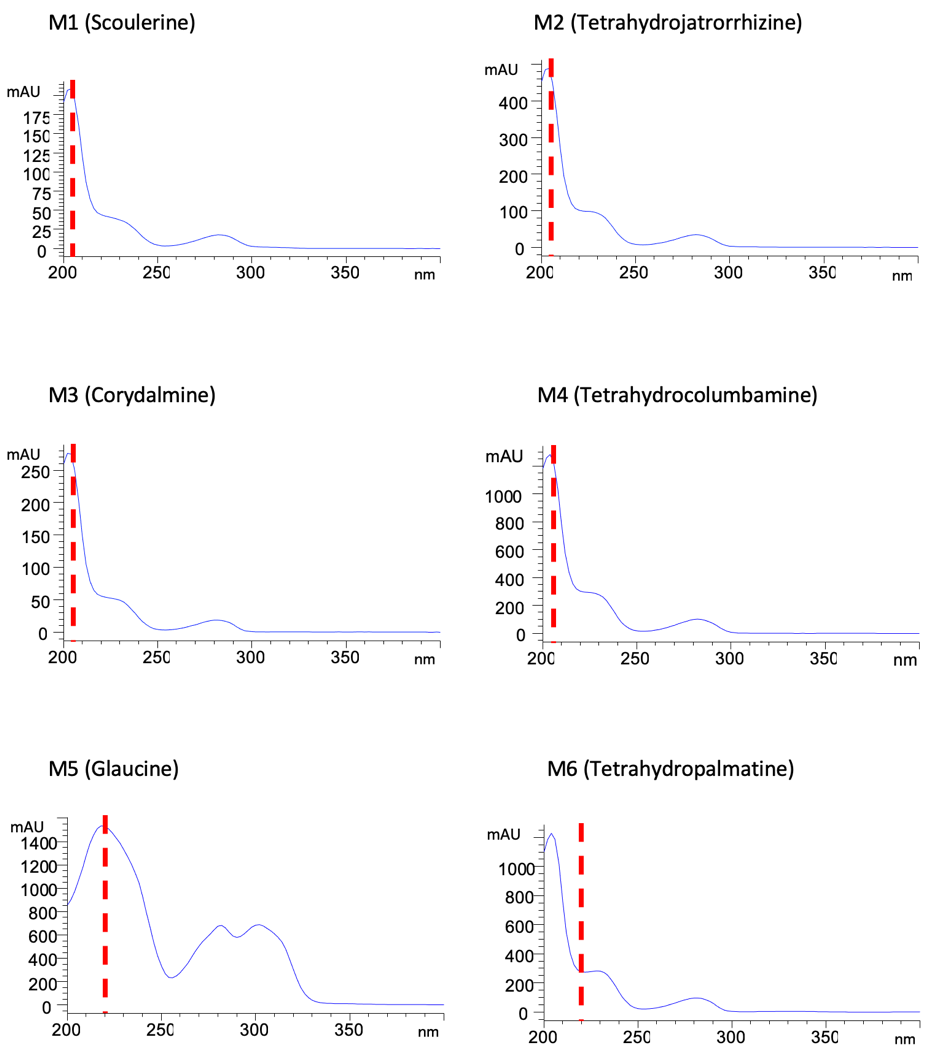

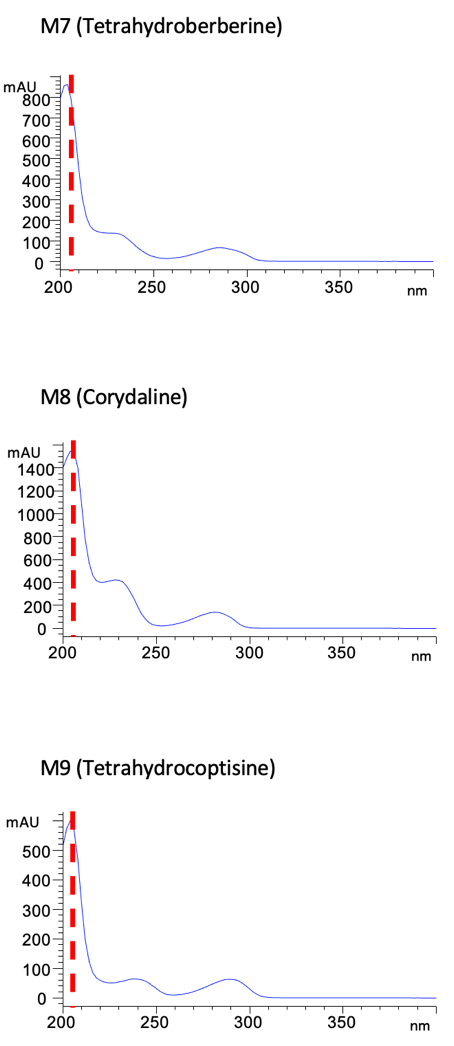


**Supplementary Figure 1.** UV/Vis spectra of alkaloids of the WCX methanol eluate. The dashed red line indicates the absorbance at 205 nm or 220 nm, respectively, that was used for quantification of the alkaloids.


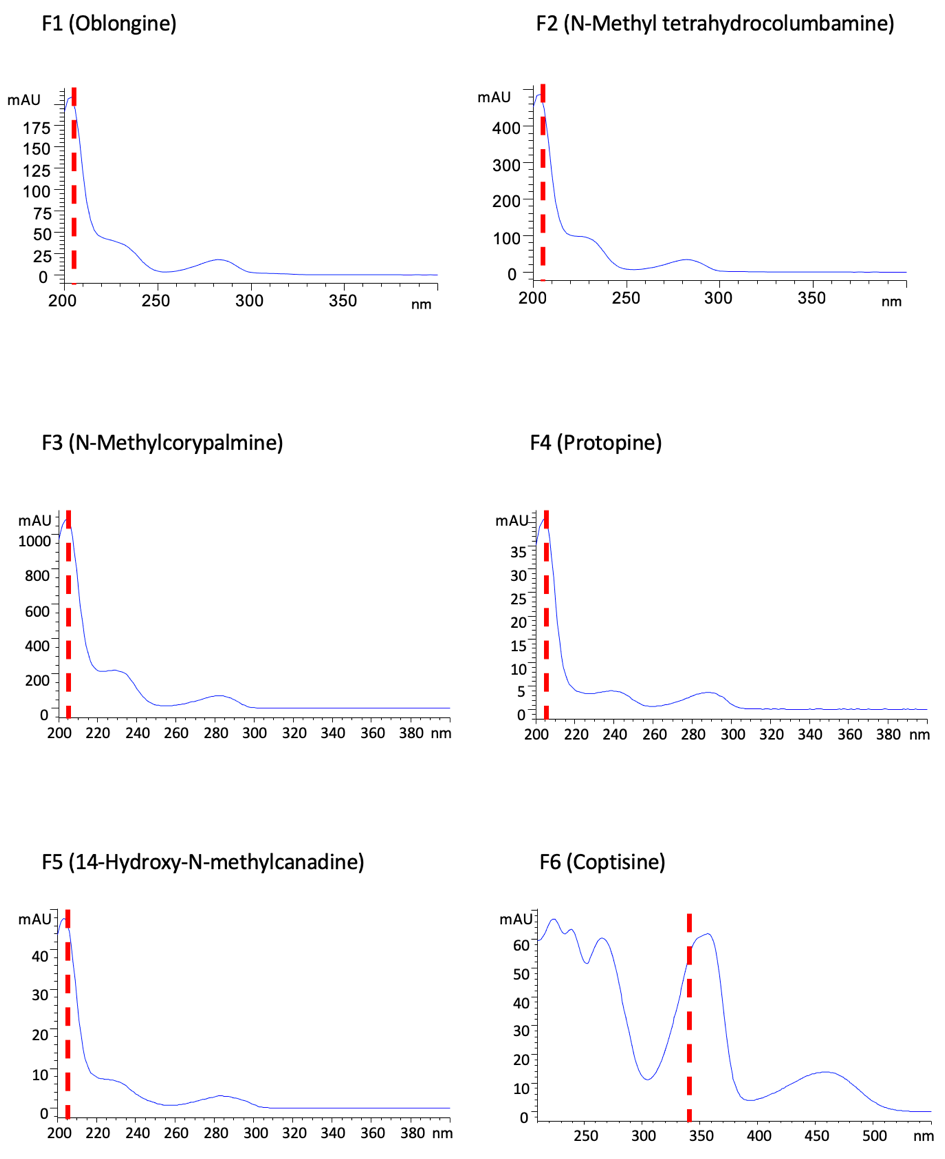

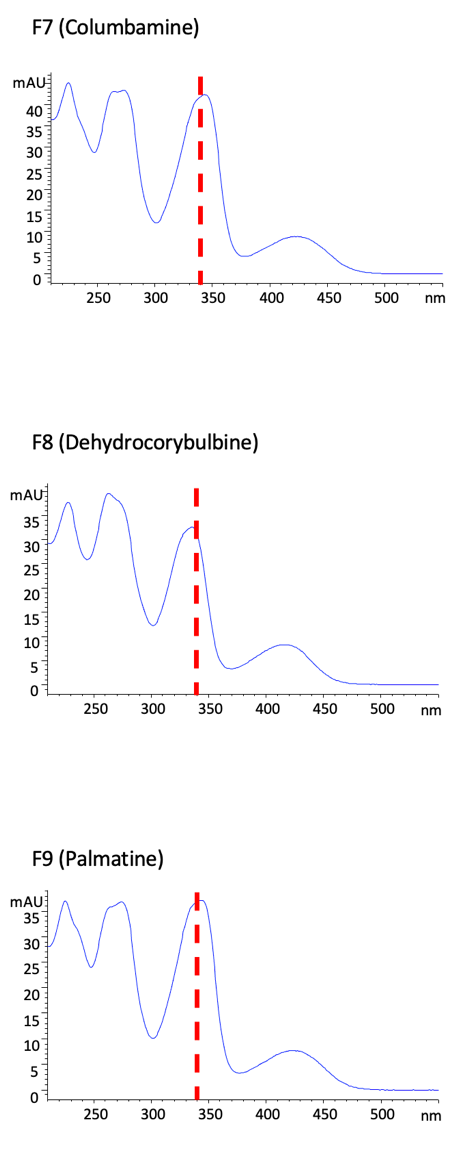


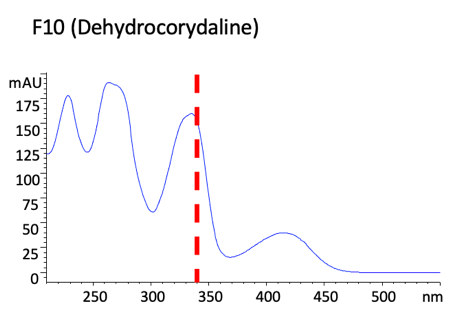


**Supplementary Figure 2.** UV/Vis spectra of alkaloids of the WCX formic acid eluate. The dashed red line indicates the absorbance at 205 nm or 340 nm, respectively, that was used for quantification of the alkaloids.


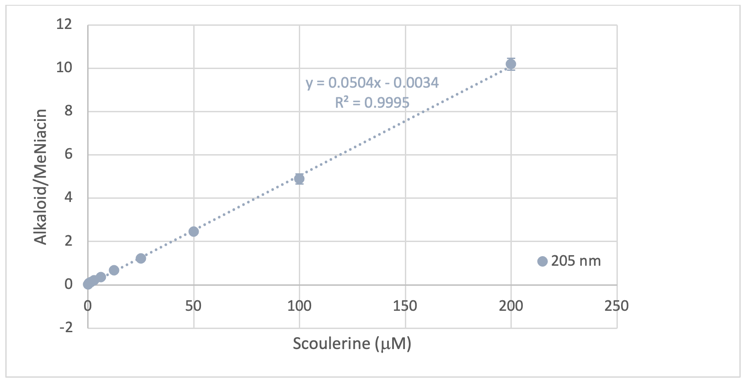

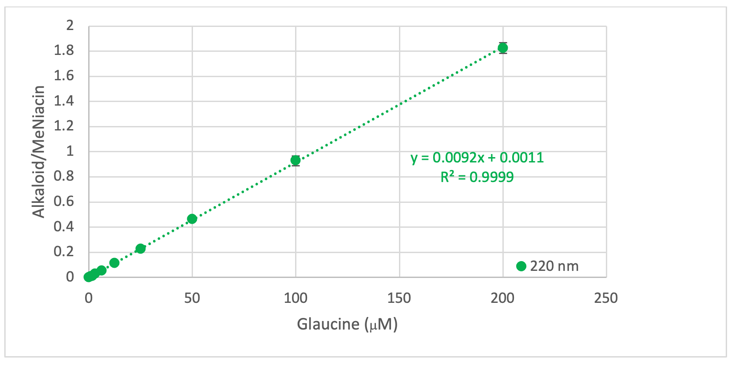


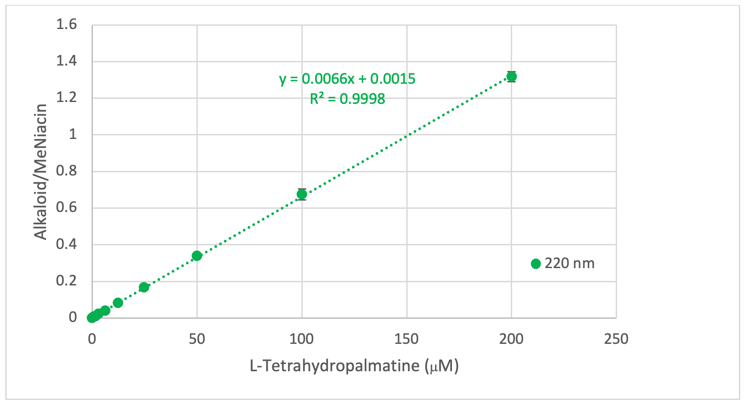

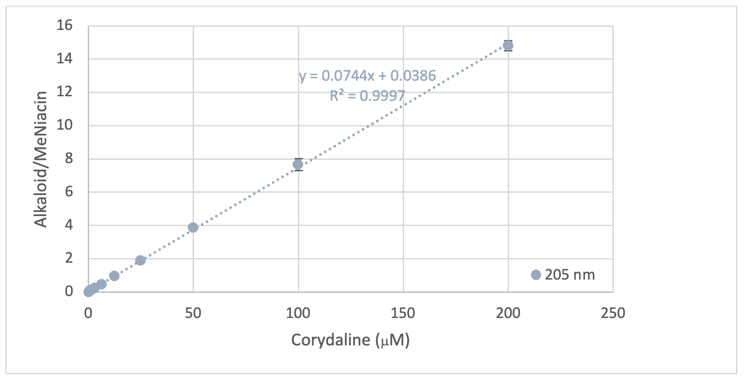

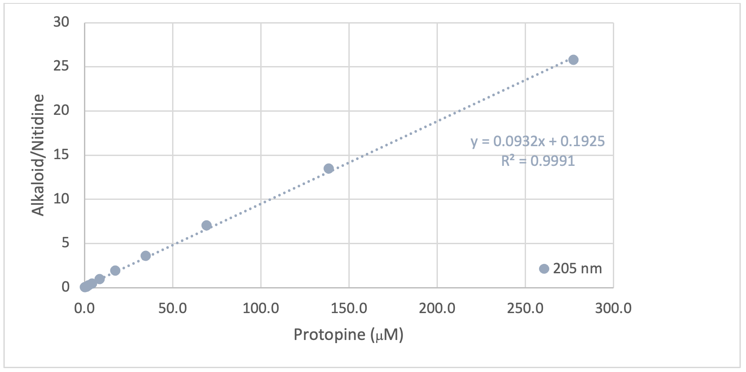

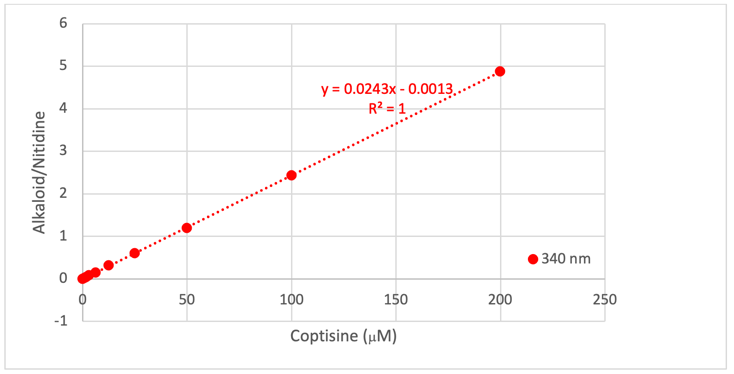

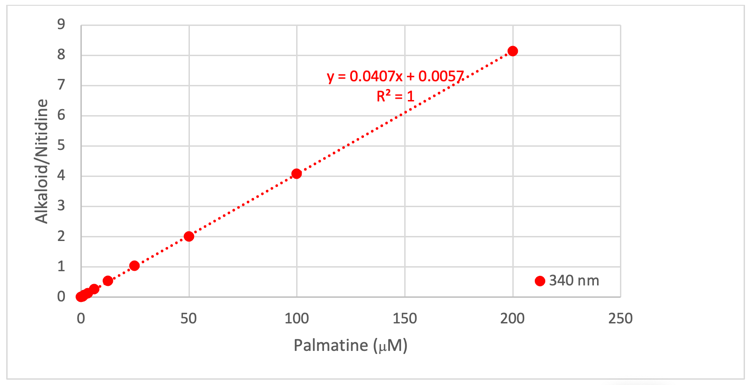


**Supplementary Figure 3.** Calibration curves for the quantification of alkaloids.

## Supplementary Tables

**Supplementary Table 1.** HR-MS data for alkaloids M1-M9 and F1-F10.

| alkaloid | Exact mass calculated | Exact mass observed | Δ (ppm) |
| --- | --- | --- | --- |
| scoulerine (M1) | 328.1549 | 328.15364 | 3.8 |
| tetrahydrojatrorrhizine (M2) | 342.1705 | 342.16931 | 3.5 |
| corydalmine (M3) | 342.1705 | 342.16937 | 3.3 |
| tetrahydrocolumbamine (M4) | 342.1705 | 342.16924 | 3.7 |
| glaucine (M5) | 356.1862 | 356.18482 | 3.9 |
| tetrahydropalmatine (M6) | 356.1862 | 356.18471 | 4.2 |
| tetrahydroberberine (M7) | 340.1549 | 340.15368 | 3.6 |
| corydaline (M8) | 370.2018 | 370.20070 | 3.0 |
| tetrahydrocoptisine (M9) | 324.1236 | 324.12236 | 3.8 |
| oblongine (F1) | 314.1751 | 314.17436 | 2.4 |
| N-methyl tetrahydrocolumbamine (F2) | 356.1856 | 356.18486 | 2.1 |
| N-methyl corypalmine (F3) | 356.1856 | 356.18474 | 2.4 |
| protopine (F4) | 354.1341 | 354.13369 | 1.2 |
| 14-hydroxy-N-methyl canadine (F5) | 370.1649 | 370.16452 | 1.0 |
| coptisine (F6) | 320.0917 | 320.09122 | 1.5 |
| columbamine (F7) | 338.1387 | 338.13792 | 2.3 |
| dehydrocorybulbine (F8) | 352.1543 | 352.15368 | 1.8 |
| palmatine (F9) | 352.1543 | 352.15371 | 1.7 |
| dehydrocorydaline (F10) | 366.1700 | 366.16935 | 1.8 |

**Supplementary Table 2.** NMR spectroscopic data (600 MHz, CD_3_CN) for scoulerine (M1).

| position | δ_C_, type^a^ | δ_H_ (*J* in Hz) | COSY | HMBC^b^ | NOESY |  |
| --- | --- | --- | --- | --- | --- | --- |
| 1 | 111.7, CH | 6.74, s |  | 3, 4a, 14 | 13_b_, 14 |  |
| 2 | 144.6, C | - |  |  |  |  |
| 3 | 145.2, C | - |  |  |  |  |
| 4 | 111.3, CH | 6.67, s |  | 2, 14a | 5_a_, 5_b_, 3-OCH_3_ |  |
| 4a | 126.1, C | - |  |  |  |  |
| 5_a_ | 28.9, CH_2_ | 2.97, m | 5_b_, 6_a_, 6_b_ | 4a, 6 | 4, 5_b_ |  |
| 5_b_ |  | 2.62, d (12.7) | 5_a_, 6_a_ | 14 | 4, 5_a_ |  |
| 6_a_ | 51.4, CH_2_ | 2.51, dt (3.5, 11.6) | 6_b_, 5_a_, 5_b_ | 14 | 6_b_, 8_b_, 14 |  |
| 6_b_ |  | 3.13, ddd (1.9, 5.3, 11.2) | 5_a_ 6_a_ | 4a, 14 | 6_a_, 8_a_ |  |
| 8_a_ | 53.3, CH_2_ | 4.07, d (15.6) | 8_b_ | 8a, 12a, 14 | 6_b_, 8_b_ |  |
| 8_b_ |  | 3.34, d (15.6) | 8_a_ | 6, 8a | 8_a_ |  |
| 8a | 121.6, C | - |  |  |  |  |
| 9 | 142.0, C | - |  |  |  |  |
| 10 | 144.6, C | - |  |  |  |  |
| 11 | 109.7, CH | 6.79, d (8.3) | 12 | 10-OCH_3_ | 12, 10-OCH_3_ |  |
| 12 | 119.1, CH | 6.63, d (8.2) | 11 | 11, 13 | 11, 13_b_ |  |
| 12a | 128.3, C | - |  |  |  |  |
| 13_a_ | 36.1, CH_2_ | 2.59, dd (4.4, 15.8) | 14, 13_b_ | 12a, 14 | 13_b_ |  |
| 13_b_ |  | 3.23, dd (3.6, 15.8) | 14, 13_a_ | 12a | 1, 12, 13_a_ |  |
| 14 | 59.2, CH | 3.39, dd (3.2, 11.3) | 13_b_, 13_a_ |  | 1, 6_a_ |  |
| 14a | 130.5, C | - |  |  |  |  |
| 3-OCH_3_ | 55.8, CH_3_ | 3.81, s |  | 3 | 4 |  |
| 10-OCH_3_ | 55.8, CH_3_ | 3.81, s |  | 10 | 11 |  |

^a^δC and type were determined from HSQC and HMBC experiments.

^b^from proton (position) to the indicated carbon(s).

**Supplementary Table 3.** NMR spectroscopic data (600 MHz, CD_3_OD) for tetrahydrojatrorrhizine (M2).

| position | δ_C_, type^a^ | δ_H_ (*J* in Hz) | COSY | HMBC^b^ | NOESY |  |
| --- | --- | --- | --- | --- | --- | --- |
| 1 | 108.5, CH | 6.86, s |  | 3, 4a, 2-OCH_3_ | 13, 2-OCH_3_ |  |
| 2 | 146.5, C | - |  |  |  |  |
| 3 | 145.7, C | - |  |  |  |  |
| 4 | 114.5, CH | 6.59, s |  | 2, 5, 14a |  |  |
| 4a | 125.6, C | - |  |  |  |  |
| 5_a_ | 27.1, CH_2_ | 3.08, m | 5_b_, 6_a_, 6_b_ |  | 5_b_ |  |
| 5_b_ |  | 2.73, d (15.4) | 5_a_, 6_a_, 6_b_ |  | 5_a_ |  |
| 6_a_ | 51.2, CH_2_ | 3.36, m | 5_a_, 5_b_, 6_b_ |  | 6_b_ |  |
| 6_b_ |  | 2.86, m | 6_a_, 5_a_, 5_b_ |  | 6_a_ |  |
| 8_a_ | 52.9, CH_2_ | 4.33, d (15.7) | 8_b_ | 8a | 8_b_ |  |
| 8_b_ |  | 3.74, d (16.0) | 8_a_ |  | 8_a_ |  |
| 8a | 125.2, C | - |  |  |  |  |
| 9 | 145.1, C | - |  |  |  |  |
| 10 | 150.5, C | - |  |  |  |  |
| 11 | 111.7, CH | 6.94, d (8.4) | 12 | 9, 12a | 10-OCH_3_ |  |
| 12 | 123.8, CH | 6.97, d (8.4) | 11 | 10, 13 |  |  |
| 12a | 126.4, C | - |  |  |  |  |
| 13_a_ | 34.6, CH_2_ | 3.52, dd (4.1, 16.3) | 13_b_ |  | 13_b_ |  |
| 13_b_ |  | 2.83, m | 13_a_ |  | 13_a_ |  |
| 14 | 67.5, CH | 4.05, dd (6.8, 7.1) |  |  |  |  |
| 14a | 126.4, C | - |  |  |  |  |
| 2-OCH_3_ | 59.3, CH_3_ | 3.85, s |  | 2 |  |  |
| 9-OCH_3_ | 55.1, CH_3_ | 3.84, s |  | 9 |  |  |
| 10-OCH_3_ | 55.1, CH_3_ | 3.83, s |  | 10 |  |  |

^a^δC and type were determined from HSQC and HMBC experiments.

^b^from proton (position) to the indicated carbon(s).

**Supplementary Table 4.** NMR spectroscopic data (600 MHz, CD_3_OD) for corydalmine (M3)

| position | δ_C_, type^a^ | δ_H_ (*J* in Hz) | COSY | HMBC^b^ | NOESY |  |
| --- | --- | --- | --- | --- | --- | --- |
| 1 | 109.0, CH | 6.88, s |  | 3, 4a, 14 | 13_a_, 14, 2-OCH_3_ |  |
| 2 | 148.1, C | - |  |  |  |  |
| 3 | 148.1, C | - |  |  |  |  |
| 4 | 111.5, CH | 6.73, s |  | 2, 5, 14a | 5_a_, 5_b_, 3-OCH_3_ |  |
| 4a | 126.1, C | - |  |  |  |  |
| 5_a_ | 27.6, CH_2_ | 3.12, m | 5_b_, 6_a_ |  | 4, 5_b_ |  |
| 5_b_ |  | 2.77, m | 5_a_ |  | 4, 5_a_ |  |
| 6_a_ | 51.1, CH_2_ | 3.32, m | 5_a_, 6_b_ |  | 6_b_, 8_a_ |  |
| 6_b_ |  | 2.79, m | 6_a_ | 4a, 14 | 6_a_, 8_b_ |  |
| 8_a_ | 53.3, CH_2_ | 4.28, d (15.6) | 8_b_ | 8a, 9, 14 | 6_b_, 8_b_ |  |
| 8_b_ |  | 3.67, d (16.2) | 8_a_ | 8a | 6_a_, 8a, 14, 9-OCH_3_ |  |
| 8a | 126.4, C | - |  |  |  |  |
| 9 | 143.6, C | - |  |  |  |  |
| 10 | 147.8, C | - |  |  |  |  |
| 11 | 115.3, CH | 6.75, d (8.2) | 12 | 9, 12a | 12 |  |
| 12 | 124.0, CH | 6.83, d (8.2) | 11 | 8a, 10, 13 | 11, 13_a_ |  |
| 12a | 125.4, C | - |  |  |  |  |
| 13_a_ | 34.8, CH_2_ | 3.46, dd (4.0, 16.5) | 13_b_, 14 | 12a | 1, 12, 13_b_, 14 |  |
| 13_b_ |  | 2.78, m | 13_a_, 14 | 8a | 13_a_, 14 |  |
| 14 | 59.6, CH | 3.75, d (12.6) | 13_a_, 13_b_ |  | 1, 8_b_, 13_a_, 13_b_ |  |
| 14a | 128.4, C | - |  |  |  |  |
| 2-OCH_3_ | 55.2, CH_3_ | 3.83, s |  | 2 | 1 |  |
| 3-OCH_3_ | 55.2, CH_3_ | 3.81, s |  | 3 | 4 |  |
| 9-OCH_3_ | 59.0, CH_3_ | 3.81, s |  | 9 |  |  |

^a^δC and type were determined from HSQC and HMBC experiments.

^b^from proton (position) to the indicated carbon(s).

**Supplementary Table 5.** NMR spectroscopic data (600 MHz, CDCl_3_/CD_3_OD 2:1; locked and referenced on CD_3_OD) for tetrahydrocolumbamine (M4).

| position | δ_C_, type^a^ | δ_H_ (*J* in Hz) | COSY | HMBC^b^ | NOESY |  |
| --- | --- | --- | --- | --- | --- | --- |
| 1 | 111.7, CH | 6.72, s | 14 | 3, 4a, 14 | 13_a_, 13_b_, 14 |  |
| 2 | 144.2, C | - |  |  |  |  |
| 3 | 146.2, C | - |  |  |  |  |
| 4 | 111.1, CH | 6.57, s | 5_a_, 5_b_ | 2, 5, 14a | 5_a_, 5_b_, 3-OCH_3_ |  |
| 4a | 125.2, C | - |  |  |  |  |
| 5_a_ | 28.4, CH_2_ | 3.06, m | 4, 5_b_ |  | 4, 5_b_ |  |
| 5_b_ |  | 2.64, m | 4, 5_a_ | 4 | 4, 5_a_ |  |
| 6_a_ | 51.6, CH_2_ | 3.17, m | 6_b_ | 5 | 6_b_, 8_a_ |  |
| 6_b_ |  | 2.61, m | 6_a_ | 4a | 6_a_, 8_b_ |  |
| 8_a_ | 53.8, CH_2_ | 4.16, d (15.8) | 8_b_ | 8a, 9, 14 | 6_a_, 8_b_ |  |
| 8_b_ |  | 3.48, d (16.2) | 8_a_ | 8a | 6_b_, 8_a_ |  |
| 8a | 127.8, C | - |  |  |  |  |
| 9 | 144.7, C | - |  |  |  |  |
| 10 | 150.2, C | - |  |  |  |  |
| 11 | 111.1, CH | 6.85, d (8.4) | 12 | 9, 12a | 12, 10-OCH_3_ |  |
| 12 | 124.1, CH | 6.78, d (8.4) | 11, 13_b_ | 10, 12a, 13 | 11, 13_a_, 13_b_ |  |
| 12a | 127.4, C | - |  |  |  |  |
| 13_a_ | 35.5, CH_2_ | 3.24, dd (1.8, 16.1) | 13_b_, 14 |  | 1, 12, 13_b_, 14 |  |
| 13_b_ |  | 2.76, dd (11.5, 16.0) | 12, 13_a_, 14 | 8a | 1, 12, 13_a_, 14 |  |
| 14 | 59.2, CH | 3.51, dd (3.4, 11.6) | 1, 13_a_, 13_b_ | 6 | 1, 13_a_, 13_b_ |  |
| 14a | 129.5, C | - |  |  |  |  |
| 3-OCH_3_ | 55.7, CH_3_ | 3.81, s |  | 3 | 4 |  |
| 9-OCH_3_ | 60.1, CH_3_ | 3.79, s |  | 9 |  |  |
| 10-OCH_3_ | 55.7, CH_3_ | 3.81, s |  | 10 | 11 |  |

^a^δC and type were determined from HSQC and HMBC experiments.

^b^from proton (position) to the indicated carbon(s).

**Supplementary Table 6.** NMR spectroscopic data (600 MHz, CD_3_OD) for tetrahydroberberine (M7).

| position | δ_C_, type^a^ | δ_H_ (*J* in Hz) | COSY | HMBC^b^ | NOESY |  |
| --- | --- | --- | --- | --- | --- | --- |
| 1 | 105.1, CH | 6.82, s | 14 | 3, 4a, 14 | 15, 13_a_ |  |
| 2 | 146.6, C | - |  |  |  |  |
| 3 | 146.6, C | - |  |  |  |  |
| 4 | 107.7, CH | 6.60, s |  | 2, 5, 14a | 15, 5_a_, 5_b_ |  |
| 4a | 127.0, C | - |  |  |  |  |
| 5_a_ | 28.3, CH_2_ | 3.07, m | 5_b_, 6_a_, 6_b_ |  | 4, 5_b_, 6_a_ |  |
| 5_b_ |  | 2.71, m | 5_a_ | 4, 4a | 4, 5_a_, 6_a_ |  |
| 6_a_ | 51.1, CH_2_ | 2.23, m | 5_a_, 6_b_ |  | 5_a_, 5_b_, 8_a_ |  |
| 6_b_ |  | 3.13, dd (3.9, 11.6) | 6_a_, 5_a_ |  | 8_b_, 14 |  |
| 8_a_ | 53.3, CH_2_ | 4.22, d (15.7) | 8_b_ | 9, 12a, 14 | 6_a_, 8_b_ |  |
| 8_b_ |  | 3.56, d (15.7) | 8_a_ | 6, 12a | 6_b_, 8_a_, 14 |  |
| 8a | ^c^, C | - |  |  |  |  |
| 9 | 144.9, C | - |  |  |  |  |
| 10 | 150.5, C | - |  |  |  |  |
| 11 | 111.4, CH | 6.90, d (8.4) |  | 9 | 10-OCH_3_ |  |
| 12 | 123.7, CH | 6.93, d (8.4) | 13_b_ | 10, 13 | 13_a_ |  |
| 12a | 127.0, C | - |  |  |  |  |
| 13_a_ | 35.2, CH_2_ | 2.38, dd (4.0, 16.2) | 14, 13_b_ |  | 1, 12, 13_b_ |  |
| 13_b_ |  | 2.75, dd (11.6, 16.1) | 12, 14, 13_a_ |  | 13_a_ |  |
| 14 | 59.7, CH | 3.62, dd (3.1, 11.2) | 13_a_, 13_b_ |  | 6_b_ |  |
| 14a | 129.6, C | - |  |  |  |  |
| 15 | 101.0, CH_2_ | 5.89, q (1.2, 0.7) |  |  | 1, 4 |  |
| 9-OCH_3_ | 59.2, CH_3_ | 3.81, s |  |  |  |  |
| 10-OCH_3_ | 55.0, CH_3_ | 3.82, s |  |  |  |  |

^a^δC and type were determined from HSQC and HMBC experiments.

^b^from proton (position) to the indicated carbon(s).

^c^ signal expected but not observed in HSQC or HMBC experiments.

**Supplementary Table 7.** NMR spectroscopic data (600 MHz, CD_3_OD) for tetrahydrocoptisine (M9).

| position | δ_C_, type^a^ | δ_H_ (*J* in Hz) | COSY | HMBC^b^ | NOESY |  |
| --- | --- | --- | --- | --- | --- | --- |
| 1 | 105.0, CH | 6.82, s | 13_b_, 14 | 3, 4a, 14 | 13_a_, 14 |  |
| 2 | 146.5, C | - |  |  |  |  |
| 3 | 146.5, C | - |  |  |  |  |
| 4 | 107.8, CH | 6.60, s | 5_a_, 5_b_ | 2, 5, 14a | 5_a_, 5_b_ |  |
| 4a | 127.0, C | - |  |  |  |  |
| 5_a_ | 28.3, CH_2_ | 3.06, m | 4, 5_b_, 6_a_ |  | 4, 5_b_, 6_a_ |  |
| 5_b_ |  | 2.71, m | 4, 5_a_, 6_a_ |  | 4, 5_a_ |  |
| 6_a_ | 50.9, CH_2_ | 3.21, m | 5_a_, 5_b_ |  | 5_b_, 6_b_, 8_a_ |  |
| 6_b_ |  | 2.69, m |  |  | 6_a_ |  |
| 8_a_ | 52.2, CH_2_ | 4.10, d (15.4) | 8_b_ | 8a, 9, 14 | 6_a_, 8_b_ |  |
| 8_b_ |  | 3.58, d (15.5) | 8_a_ | 6, 8a, 9 | 8_a_, 14 |  |
| 8a | 115.4, C | - |  |  |  |  |
| 9 | 144.4, C | - |  |  |  |  |
| 10 | 144.4, C | - |  |  |  |  |
| 11 | 106.6, CH | 6.70, s^c^ |  | 10, 12a |  |  |
| 12 | 121.0, CH | 6.70, s^c^ |  | 8a, 10, 13 | 13_a_, 13_b_ |  |
| 12a | 127.6, C | - |  |  |  |  |
| 13_a_ | 35.3, CH_2_ | 3.40, dd (3.7, 16.4) | 13_b_, 14 |  | 1, 12, 13_b_, 14 |  |
| 13_b_ |  | 2.75, m | 1, 13_a_, 14 |  | 12, 13_a_ |  |
| 14 | 59.7, CH | 3.67, m | 1, 13_a_, 13_b_ |  | 1, 8_b_, 13_a_ |  |
| 14a | 129.6, C | - |  |  |  |  |
| 15 | 101.1, CH_2_ | 5.89, q (1.2, 0.9) |  |  |  |  |
| 16 | 101.4, CH_2_ | 5.94, dd (1.2, 13.4) |  | 9, 10 |  |  |

^a^δC and type were determined from HSQC and HMBC experiments.

^b^from proton (position) to the indicated carbon(s).

^c^coinciding signals.

**Supplementary Table 8.** NMR spectroscopic data (600 MHz, CD_3_OD) for oblongine (F1).

| position | δ_C_, type^a^ | δ_H_ (*J* in Hz) | COSY | HMBC^b^ |  |
| --- | --- | --- | --- | --- | --- |
| 1 | 69.1, CH | 5.04, m | 9_a,_ 9_b_ | 8a, 10 |  |
| 2 | - | - |  |  |  |
| 3_a_ | 54.0, CH_2_ | 3.73, dt (6.5, 12.6) | 3_b_, 4_a_, 4_b_ | 4 |  |
| 3_b_ |  | 3.43, m | 3_a_ | 4 |  |
| 4_a_ | 22.4, CH_2_ | 3.12-3.18, m | 3_b_, 3_a_ | 3 |  |
| 4_b_ |  | 2.98, dd (5.9, 18.0) | 3_a_ |  |  |
| 4a | 120.6, C | - |  |  |  |
| 5 | 118.9, CH | 6.75, d (8.4) | 6 | 4, 7, 8a |  |
| 6 | 111.8, CH | 7.01, d (8.4) | 5 | 4a, 8 |  |
| 7 | 146.3, C | - |  |  |  |
| 8 | 143.0, C | - |  |  |  |
| 8a | 118.8, C | - |  |  |  |
| 9_a_ | 36.1, CH_2_ | 3.41, m | 1 | 11 |  |
| 9_b_ |  | 3.23, dd (2.6, 16.2) | 1 | 15 |  |
| 10 | 127.5, C | - |  |  |  |
| 11, 15^c^ | 130.0, CH | 7.04, d (8.5) | 12, 14 | 9, 11, 13, 15 |  |
| 12, 14 ^c^ | 115.2, CH | 6.70, d (8.5) | 11, 15 | 10, 12, 14 |  |
| 13 | 156.5, C | - |  |  |  |
| 16 | 52.7, CH_3_ | 3.15, s |  | 1 |  |
| 17 | 50.4, CH_3_ | 3.06, s |  | 1 |  |
| 7-OCH_3_ | 55.3, CH_3_ | 3.87, s |  | 7 |  |

^a^δC and type were determined from HSQC and HMBC experiments.

^b^from proton (position) to the indicated carbon(s).

^c^ signals exchangeable.

**Supplementary Table 9.** NMR spectroscopic data (600 MHz, CD_3_CN) for N-methyl tetrahydrocolumbamine (F2).

| position | δ_C_, type^a^ | δ_H_ (*J* in Hz) | COSY | HMBC^b^ | NOESY |  |
| --- | --- | --- | --- | --- | --- | --- |
| 1 | 113.0, CH | 7.13, br s | 14 | 3, 4a, 14 | 13_a_, 13_b_, 14 |  |
| 2 | 149.8, C | - |  |  |  |  |
| 3 | 149.0, C | - |  |  |  |  |
| 4 | 111.6, CH | 6.70, s | 5_a_, 5_b_, 3-OCH_3_ | 2, 5, 14a | 5_a_, 5_b_, 3-OCH_3_ |  |
| 4a | 117.8, C | - |  |  |  |  |
| 5_a_ | 23.0, CH_2_ | 3.24, m | 4, 5_b_, 6_a_, 6_b_ | 4a, 6 | 4, 5_b_, 6_a_, 15 |  |
| 5_b_ |  | 3.01, dd (3.9, 18.1) | 4, 5_a_, 6_b_ |  | 4, 5_a_ |  |
| 6_a_ | 62.1, CH_2_ | 3.83, m^c^ | 6_b_, 5_a_ | 4a, 5, 14 | 5_a_, 6_b_, 8_a_ |  |
| 6_b_ |  | 3.73, dt (5.0, 12.5) | 6_a_, 5_a_, 5_b_ | 5, 15 | 6_a_, 8_b_ |  |
| 8_a_ | 61.5, CH_2_ | 4.69, d (15.9) | 8_b_ | 8a, 9, 12a, 14, 15 | 6_a_, 8_b_, 15 |  |
| 8_b_ |  | 4.52, d (16.0) | 8_a_, 15 | 6, 8a, 15 | 6_b_, 8_a_, 14, 9-OCH_3_ |  |
| 8a | 120.2, C | - |  |  |  |  |
| 9 | 145.5, C | - |  |  |  |  |
| 10 | 151.1, C | - |  |  |  |  |
| 11 | 113.4, CH | 7.06, d (8.5) | 12 | 9, 12a | 10-OCH_3_ |  |
| 12 | 124.5, CH | 7.08, d (8.6) | 11, 13_a_, 13_b_ | 8a, 10, 13 |  |  |
| 12a | 123.0, C | - |  |  |  |  |
| 13_a_ | 28.2, CH | 2.91, dd (12.4, 17.9) | 12, 13_b_, 14 | 12a, 14 | 1, 13_b_, 15 |  |
| 13_b_ |  | 3.84, m^c^ | 12, 13_a_, 14 | 14a | 1, 13_a_, 14 |  |
| 14 | 66.2, C | 4.75, dd (4.8, 12.3) | 13_b_, 13_a_ | 8, 14a, 15 | 13_b_, 1 |  |
| 14a | 121.7, C | - |  |  |  |  |
| 15 | 38.9, CH_3_ | 2.78, s | 8_b_ | 6, 8, 14 | 5_a_, 8_a_, 13_a_ |  |
| 3-OCH_3_ | 55.4, CH_3_ | 3.78, s | 4 | 3 |  |  |
| 9-OCH_3_ | 60.2, CH_3_ | 3.83, s^c^ |  | 9 |  |  |
| 10-OCH_3_ | 55.6, CH_3_ | 3.85, s^c^ |  | 10 |  |  |

^a^δC and type were determined from HSQC and HMBC experiments.

^b^from proton (position) to the indicated carbon(s).

^c^ signals overlap; the shift of the methylene protons was estimated from the COSY experiment.

**Supplementary Table 10.** NMR spectroscopic data (600 MHz, CD_3_CN) for N-methyl corypalmine (F3).

| position | δ_C_, type^a^ | δ_H_ (*J* in Hz) | COSY | HMBC^b^ | NOESY |  |
| --- | --- | --- | --- | --- | --- | --- |
| 1 | 109.4, CH | 6.75, s |  | 3, 4a, 14 | 13_b_, 2-OCH_3_ |  |
| 2 | 148.6, C | - |  |  |  |  |
| 3 | 151.2, C | - |  |  |  |  |
| 4 | 115.7, CH | 6.71, s |  | 2, 5, 14a |  |  |
| 4a | 122.6, C | - |  |  |  |  |
| 5_a_ | 23.0, CH_2_ | 3.24, m | 5_b_, 6_a_, 6_b_ |  | 5_b_,15 |  |
| 5_b_ |  | 2.98, m | 5_a_, 6_b_ |  | 5_a_ |  |
| 6_a_ | 62.0, CH_2_ | 3.82, m^c^ | 5_a_ |  | 6_b_, 8_a_ |  |
| 6_b_ |  | 3.68, m | 5_a_, 5_b_ |  | 6a, 8_b_, 14 |  |
| 8_a_ | 61.4, CH_2_ | 4.71, d (15.9) | 8_b_ | 9, 14 | 6_a_, 8_b_ |  |
| 8_b_ |  | 4.49, d (16.0) | 8_a_, 15 | 9, 14, 15 | 6_b_, 8_a_, 14, 15 |  |
| 8a | 120.4, C | - |  |  |  |  |
| 9 | 148.7, C | - |  |  |  |  |
| 10 | 145.3, C | - |  |  |  |  |
| 11 | 113.5, CH | 7.08, d (8.5) | 12 | 12a | 10-OCH_3_ |  |
| 12 | 124.4, CH | 7.10, d (8.6) | 11 |  | 13_b_ |  |
| 12a | 122.4, C | - |  |  |  |  |
| 13_a_ | 28.4, CH | 2.95, dd (12.4, 17.7) | 14, 13_b_ | 12a, 14 | 13_b_, 15 |  |
| 13_b_ |  | 3.90, d (17.7) | 14, 13_a_ | 12a | 1, 12, 12a, 13_a_ |  |
| 14 | 66.5, C | 4.76, dd (4.6, 12.3) | 13_a_, 13_b_ |  | 6_b_, 8_b_ |  |
| 14a | 117.4, C | - |  |  |  |  |
| 15 | 38.8, CH_3_ | 2.80, s | 8_b_ |  |  |  |
| 2-OCH_3_ | 55.6, CH_3_ | 3.86, s |  |  | 1 |  |
| 9-OCH_3_ | 55.8, CH_3_ | 3.83, s^c^ |  |  |  |  |
| 10-OCH_3_ | 60.3, CH_3_ | 3.82, s^c^ |  |  |  |  |

^a^δC and type were determined from HSQC and HMBC experiments.

^b^from proton (position) to the indicated carbon(s).

^c^ signals overlap; the shift of the methylene protons was estimated from the COSY experiment.

**Supplementary Table 11.** NMR spectroscopic data (600 MHz, CD_3_OD) for 14-hydroxy-N-methyl canadine (F5).

| position | δ_C_, type^a^ | δ_H_ (*J* in Hz) | COSY | HMBC^b^ |  |
| --- | --- | --- | --- | --- | --- |
| 1 | 105.5, CH | 7.21, s |  | 3, 4a, 14 |  |
| 2 | 148.1, C | - |  |  |  |
| 3 | 148.8, C | - |  |  |  |
| 4 | 107.9, CH | 6.79, |  | 2, 5, 14a |  |
| 4a | 123.9, C | - |  |  |  |
| 5_a_ | 23.5, CH_2_ | 3.37, m | 6_a_, 6_b_ |  |  |
| 5_b_ |  | 3.10, m | 6_a_ |  |  |
| 6_a_ | 54.1, CH_2_ | 4.04, dt (5.2, 12.7) | 5_a_, 5_b_, 6_b_ |  |  |
| 6_b_ |  | 3.64, m | 5_a_, 6_a_ | 4a |  |
| 8_a_ | 53.1, CH_2_ | 3.84, m |  |  |  |
| 8_b_ |  | 3.74, m |  |  |  |
| 8a | 121.5, C | - |  |  |  |
| 9 | 145.2, C | - |  |  |  |
| 10 | 151.2, C | - |  |  |  |
| 11 | 113.3, CH | 7.11, s |  |  |  |
| 12 | 124.7, CH | 7.11, s |  | 8a, 13 |  |
| 12a | ^c^ | - |  |  |  |
| 13_a_ | 35.0, CH_2_ | 3.95, d (17.8) | 13_b_ | 8a, 14 |  |
| 13_b_ |  | 3.41, d (17.6) | 13_s_ | 8a |  |
| 14 | 91.6, C | - |  |  |  |
| 14a | 125.4, C | - |  |  |  |
| 15 | 102.7, CH_2_ | 6.02, d (2.3) |  |  |  |
| 16 | 41.4, CH_3_ | 3.00, s |  | 14 |  |
| 9-OCH_3_ | 59.6, CH_3_ | 3.90, s |  | 9 |  |
| 10-OCH_3_ | 55.1, CH_3_ | 3.88, s |  | 10 |  |

^a^δC and type were determined from HSQC and HMBC experiments.

^b^from proton (position) to the indicated carbon(s).

^c^ a chemical shift for this carbon could not be determined.

**Supplementary Table 12.** NMR spectroscopic data (600 MHz, CD_3_CN) for coptisine (F6).

| position | δ_C_, type^a^ | δ_H_ (*J* in Hz) | COSY | HMBC^b^ | NOESY |  |
| --- | --- | --- | --- | --- | --- | --- |
| 1 | 105.1, CH | 7.52, s |  | 3, 4a | 13 |  |
| 2 | 148.5, C | - |  |  |  |  |
| 3 | 150.3, C | - |  |  |  |  |
| 4 | 108.3, CH | 6.92, s |  | 2, 5, 14a |  |  |
| 4a | 130.5, C | - |  |  |  |  |
| 5 | 26.6, CH_2_ | 3.18, t (6.4) | 6 | 4, 4a, 6, 14a | 6 |  |
| 6 | 56.0, CH_2_ | 4.76, t (6.3) | 5 | 4a, 5, 8, 14 | 5 |  |
| 8 | 143.3, CH | 9.41, s |  | 6, 9, 12a, 14 |  |  |
| 8a | 112.1, C | - |  |  |  |  |
| 9 | 144.3, C | - |  |  |  |  |
| 10 | 148.0, C | - |  |  |  |  |
| 11 | 121.2, CH | 7.83, d (8.6) |  | 9, 12a | 12 |  |
| 12 | 121.6, CH | 7.77, d (8.6) |  | 8a, 10, | 11, 13 |  |
| 12a | 132.8, C | - |  |  |  |  |
| 13 | 121.4, CH | 8.57, s |  | 8a, 12, 14 | 1, 12 |  |
| 14 | 137.5, C | - |  |  |  |  |
| 14a | 120.4, C | - |  |  |  |  |
| 15 | 103.4, CH_2_ | 6.09, s |  | 2, 3 |  |  |
| 16 | 105.6, CH_2_ | 6.42, s |  | 9, 10 |  |  |

^a^δC and type were determined from HSQC and HMBC experiments.

^b^from proton (position) to the indicated carbon(s).

**Supplementary Table 13.** NMR spectroscopic data (600 MHz, CD_3_OD) for columbamine (F7).

| position | δ_C_, type^a^ | δ_H_ (*J* in Hz) | HMBC^b^ | NOESY |  |
| --- | --- | --- | --- | --- | --- |
| 1 | 112.6 CH | 7.50, s | 3, 4a, 14 | 13 |  |
| 2 | 149.3, C | - |  |  |  |
| 3 | 153.0, C | - |  |  |  |
| 4 | 111.4, CH | 6.96, s | 2, 5, 14a | 5, 3-OCH_3_ |  |
| 4a | 128.8, C | - |  |  |  |
| 5 | 27.3, CH_2_ | 3.19, t (6.3) | 4a | 4, 6 |  |
| 6 | 56.9, CH_2_ | 4.85, t (6.4) | 5, 4a, 8 | 5, 8 |  |
| 8 | 145.3, CH | 9.68, s | 6, 12a, 14 | 6 |  |
| 8a | 124.0, C | - |  |  |  |
| 9 | 146.3, C | - |  |  |  |
| 10 | 152.3, C | - |  |  |  |
| 11 | 127.3, CH | 8.03, d (9.1) | 9, 12a | 12, 10-OCH_3_ |  |
| 12 | 123.8, CH | 7.93, d (9.1) | 8a, 10 | 11, 13 |  |
| 12a | 136.3, C | - |  |  |  |
| 13 | 120.3, CH | 8.58, s | 8a, 14 | 1, 12 |  |
| 14 | 140.6, C | - |  |  |  |
| 14a | 121.3 C | - |  |  |  |
| 3-OCH_3_ | 56.0, CH_3_ | 3.90, s | 3 | 4 |  |
| 9-OCH_3_ | 57.0, CH_3_ | 4.14, s | 9 |  |  |
| 10-OCH_3_ | 61.9, CH_3_ | 4.04, s | 10 | 11 |  |

^a^δC and type were determined from HSQC and HMBC experiments.

^b^from proton (position) to the indicated carbon(s).

**Supplementary Table 14.** NMR spectroscopic data (600 MHz, CD_3_CN) for dehydrocorybulbine (F8).

| position | δ_C_, type^a^ | δ_H_ (*J* in Hz) | HMBC^b^ | NOESY^c^ | |  |
| --- | --- | --- | --- | --- | --- | --- |
| 1 | 114.6 CH | 7.24, s | 2, 3, 4a, 14 | 15, 2-OCH_3_ | |  |
| 2 | 147.2, C | - | - |  | |  |
| 3 | 152.4, C | - | - |  | |  |
| 4 | 114.7, CH | 6.96, s | 2, 5, 14a | 5 | |  |
| 4a | 132.4, C | - | - | 4 | |  |
| 5 | 27.2, CH_2_ | 3.03, t (6.0) | 6, 4, 4a | 6 | |  |
| 6 | 57.6, CH_2_ | 4.66, t (6.0) | 5, 4a, 8 | 5, 8 | |  |
| 8 | 142.6, CH | 9.49, s | 6, 9, 12a | 6, 9-OCH_3_ | |  |
| 8a | 120.9, C | - | - |  | |  |
| 9 | 144.4, C | - | - |  | |  |
| 10 | 150.2, C | - | - |  | |  |
| 11 | 125.9, CH | 8.01, d (9.4) | 9, 12a | 10-OCH_3_ | |  |
| 12 | 120.7, CH | 8.05, d (9.4) | 8a, 10 | 15 | |  |
| 12a | 134.2, C | - | - |  | |  |
| 13 | 129.7, C | - | - |  | |  |
| 14 | 137.8, C | - | - |  | |  |
| 14a | 116.3 C | - | - |  | |  |
| 15 | 17.6, CH_3_ | 2.96, s | 12a, 13, 14 | 1, 12, 2-)CH_3_ | |  |
| 2-OCH_3_ | 56.1, CH_3_ | 3.89, s | 2 | 1, 15 | |  |
| 9-OCH_3_ | 56.8, CH_3_ | 4.12, s | 9 | 6 | |  |
| 10-OCH_3_ | 61.8, CH_3_ | 4.07, s | 10 | 11 |  | |

^a^δC and type were determined from HSQC and HMBC experiments.

^b^from proton (position) to the indicated carbon(s).

^c^NOESY spectrum was acquired in CD_3_OD.
